# Metabolic reprogramming: A novel therapeutic target in diabetic kidney disease

**DOI:** 10.3389/fphar.2022.970601

**Published:** 2022-09-02

**Authors:** Mengdi Wang, Yanyu Pang, Yifan Guo, Lei Tian, Yufei Liu, Cun Shen, Mengchao Liu, Yuan Meng, Zhen Cai, Yuefen Wang, Wenjing Zhao

**Affiliations:** Department of Nephrology, Beijing Hospital of Traditional Chinese Medicine, Capital Medical University, Beijing, China

**Keywords:** diabetic kidney disease, metabolic reprogramming, energy metabolism, glycolysis, mitochondrial oxidative phosphorylation

## Abstract

Diabetic kidney disease (DKD) is one of the most common microvascular complications of diabetes mellitus. However, the pathological mechanisms contributing to DKD are multifactorial and poorly understood. Diabetes is characterized by metabolic disorders that can bring about a series of changes in energy metabolism. As the most energy-consuming organs secondary only to the heart, the kidneys must maintain energy homeostasis. Aberrations in energy metabolism can lead to cellular dysfunction or even death. Metabolic reprogramming, a shift from mitochondrial oxidative phosphorylation to glycolysis and its side branches, is thought to play a critical role in the development and progression of DKD. This review focuses on the current knowledge about metabolic reprogramming and the role it plays in DKD development. The underlying etiologies, pathological damages in the involved cells, and potential molecular regulators of metabolic alterations are also discussed. Understanding the role of metabolic reprogramming in DKD may provide novel therapeutic approaches to delay its progression to end-stage renal disease.

## Introduction

Diabetic kidney disease (DKD) is the dominant cause of end-stage renal disease (ESRD) worldwide (Alicic et al., 2017). As the incidence and prevalence of DKD have surged significantly in consistence with the global epidemic of diabetes, it has placed large burdens on the society and the families of affected patients (Tuttle et al., 2014). Therefore, slowing the rate of progression of DKD is obviously of great importance. However, few therapies have been shown to be particularly effective. The pathological mechanisms contributing to the development of DKD are complex, with multiple factors involved. Notably, diabetes mellitus is characterized by metabolic abnormalities, such as hyperglycemia and hyperlipemia, which cause deleterious effects on the kidneys. However, strict blood glucose control has not led to positive clinical outcomes (Group, 2003; Zhang et al., 2003), nor has the available clinical management of hyperlipidemia (Haynes et al., 2014). To find effective therapies, deeper investigations into other mechanisms mediating the influence of metabolic disorders on kidney damage are needed.

The kidneys are high-energy-consuming organs (Wang et al., 2010). They require a large amount of energy to remove waste from the blood, reabsorb nutrients, balance electrolytes and fluids, maintain acid–base homeostasis, and regulate blood pressure (Bhargava and Schnellmann, 2017). Therefore, a normal and balanced energy metabolism system is particularly important for maintaining the specific structure and physiological function of kidneys (Chen et al., 2016). The metabolic process shows plasticity and can change in accordance with environmental changes. Metabolic reprogramming, also known as the “Warburg effect”, was first observed by Warburg in 1958; he found that tumor cells can synthesize adenosine-5′-triphosphate (ATP) through glycolysis even under well-oxygenated conditions (Warburg et al., 1927). In recent decades, technological advances have enabled a better understanding of energy metabolism. An increasing number of studies have confirmed the crucial role played by metabolic reprogramming in the development of chronic kidney diseases, such as renal fibrosis and autosomal dominant polycystic kidney disease (AKDKD) (Pagliarini and Podrini, 2021; Zhu et al., 2021). Recently, the metabolic alterations that drive the change from mitochondrial oxidative phosphorylation (OXPHOS) to glycolysis and its principal branches have attracted increasing interest with respect to delineating DKD mechanisms (Laustsen et al., 2013). In this review, we summarize the potential mechanism of diabetes-induced metabolic reprogramming, provide insights into the roles they play in the pathogenesis of renal cell damage, identify potential biomarkers, and discuss promising therapeutic strategies targeting metabolic reprogramming that prevent or halt renal injury in diabetes.

## Mechanism of metabolic reprogramming in diabetic kidney disease

**FIGURE 1:**
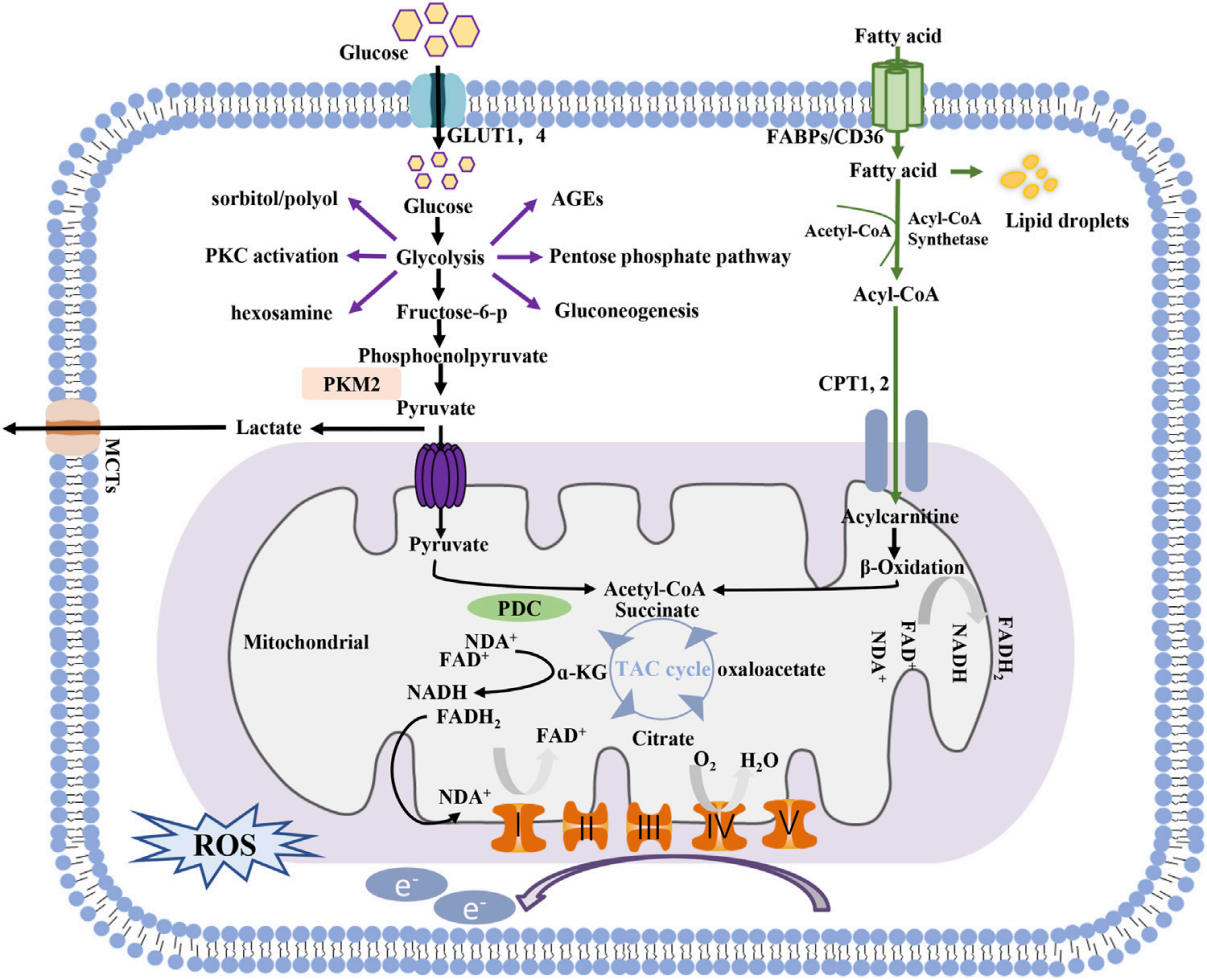
Process of energy metabolism in cells.

Glycolysis and mitochondrial oxidative phosphorylation are two main pathways of energy generation in cells. In glycolysis, one molecule of glucose is reduced to pyruvate in the cytoplasm, generating two molecules of ATP. The substrates for mitochondrial oxidative phosphorylation are more diverse than those involved in glycolysis. Pyruvate generated by glycolysis can be further shuttled into the tricarboxylic acid (TCA) cycle for OXPHOS in mitochondria, which produces an additional 36 molecules of ATP in the presence of oxygen. Free fatty acid utilization depends mainly on mitochondria, with 106 molecules of ATP generated through the complete oxidation of one molecule of palmitate. Glutamine can also be used to fuel OXPHOS in certain cells. Notably, glucose can be metabolized via side branches of glycolysis, including the advanced glycation end-product pathway, sorbitol/polyol pathway, diacylglycerol protein kinase C pathway, and hexosamine pathway, but no ATP is generated through these pathways, and ion flux under basal conditions is low (Figure 1).

Compared with glycolysis, OXPHOS is obviously more efficient for ATP generation; therefore, under normal circumstances, OXPHOS is the main source of ATP-based energy in the kidney, with a small amount deriving from glycolysis (Abe et al., 2010; Ahmad et al., 2021). However, the ATP production rate of the glycolytic pathway can be 10–100-fold faster than that of OXPHOS, and it has tremendous potential to be further enhanced in response to pathological conditions (Shiraishi et al., 2015). Diabetes, characterized by altered cellular metabolism, is thought to drive metabolic switching from oxidative phosphorylation to glycolysis or its side branches in renal cells. Although the underlying mechanisms are not fully understood, several potential etiologies leading to this process have been implicated.

### Mitochondria dysfunction

Mitochondria are double-membraned organelles that provide sites for cellular respiration and oxygen-consuming ATP production via OXPHOS. The oxidative power of mitochondria depends on substrate utilization through a series of enzymes. ATP synthase, located on the inner membrane of mitochondria, catalyzes the phosphorylation of adenosine diphosphate (ADP) to ATP. The phosphorylation process is powered by a proton-motive force formed by the action of three respiratory chain complexes named CI, CIII, and CIV, which pump protons from the inner matrix of the mitochondria into the intermembrane space. In addition to forming an efficient coupling between electron transmission and ATP generation, respiratory chain complexes provide electrons to O_2_, which generate H_2_O. Electrons in the respiratory chain are available through the action of the reducing equivalents nicotinamide adenine dinucleotide (NADH) and flavin adenine dinucleotide (FADH_2_), which are mainly generated through a sequence of enzymatically catalyzed reactions in the matrix of the mitochondria known as the tricarboxylic acid (TCA) cycle.

Recently, Sas et al. (Sas et al., 2016) found that although metabolic flux mediated through the TCA cycle was increased in the diabetic kidney cortex, neither oxygen consumption nor ATP production was increased. A metabolic switch to anaerobic glycolysis to produce energy was identified when the mitochondrial function was suppressed (Abe et al., 2010; Brinkkoetter et al., 2019), suggesting that mitochondrial dysfunction in diabetes may lead to metabolic alteration. Mitochondria are susceptible to a variety of genetic and environmental insults. In fact, mitochondrial dysfunction in DKD has been revealed through many studies, which have shown induction of mitochondrial DNA (mtDNA) mutations and deletions (reduced mtDNA stability) (Czajka et al., 2015), decreased expression of electron transport chain (ETC) complex genes and mitochondrial biogenesis (Dugan et al., 2013), a defective mitochondrial fusion–fission process, and mitophagy disorders (Higgins and Coughlan, 2014; Dai et al., 2021; Zhang et al., 2021). An increase in the uncoupling of the respiratory chain may lead to diminished ATP synthesis in diabetic mitochondria (Friederich et al., 2008). In addition, sustained hyperglycemia further induces an abnormally high proton gradient across the inner mitochondrial membrane, leading to excessive reactive oxygen species (ROS) or reactive nitrogen species (RNS) production (Friederich et al., 2008), which results in a vicious cycle by promoting mitochondrial dysfunction (Brady et al., 2006; Zorov et al., 2006, 2014; Venditti and Di Meo, 2020). Moreover, the excessive metabolic byproduct was caused by the decreased activities of mitochondria in diabetes, such as citrate succinate, fumarate, and malate, and the accumulation of these byproducts is toxic to mitochondria by inhibiting ATP synthase (Fu X. et al., 2015) or decreasing the mitochondrial membrane potential (Karlstaedt et al., 2016).

### Increased glycolytic flux

The diabetic milieu is characterized by excessive energetic substrates, including glucose, which is taken up by renal cells via glucose transporters (Elsas and Longo, 1992). Hyperglycemia enhances glucose transportation from extracellular to intracellular compartments by upregulating the expression of glucose transporters (GLUTs) or sodium–glucose cotransporters (SGLTs) (Heilig et al., 1997). Glomerular cells take up most of the excessive glucose by overexpressing transporter isoforms of GLUT1 (Weigert et al., 2003; Moutzouris et al., 2007). Mechanical stress resulting from glomerular hypertension has been shown to be another contributor to increased glycolytic flux (Lewko et al., 2005). In proximal tubule cells, SGLT2 reabsorbs glomerular-filtered glucose from the lumen of the proximal tubules on the apical side. However, despite exposure to elevated intracellular glucose, this reabsorbed glucose is not consumed during ATP production in proximal tubules under normal conditions but diffuses into the interstitial space through GLUT2 on the basolateral side and is then transported back into the bloodstream (Mather and Pollock, 2011). Therefore, in addition to elevated glucose intake, the expression of glycolytic enzymes is upregulated to enhance glucose decomposition under high-glucose (HG) conditions (Jiang et al., 2019). Recent work by Sas et al. (Sas et al., 2016) demonstrated significantly increased levels of several important glycolytic enzyme transcripts, including hexokinase, phosphofructokinase, and pyruvate kinase, in the diabetic kidney, and the protein products of these transcripts catalyze three irreversible reactions in glycolysis. However, the unchanged expression of TCA cycle pathway-related genes was observed in the study. For example, the pyruvate dehydrogenase multienzyme complex (PDC), a key regulator linking glycolysis to the TCA cycle by catalyzing pyruvate to acetyl-coenzyme A (CoA) irreversibly in mitochondria, was shown to be hyperphosphorylated and inhibited, leading to diabetic kidney injury in the presence of consistent hyperglycemia (Jeoung et al., 2006; Rardin et al., 2009; Dugan et al., 2013; Dlamini et al., 2015). Hence, more pyruvate undergoes anaerobic fermentation to lactate in a compensatory process.

When glycolytic lactic acid reaches the saturation points, some of the excess glycolytic intermediate metabolites are shunted down its side branches (Buse, 2006). For example, the polyol pathway was shown to metabolize as much as 33% of this glucose when hexokinase abundance reached the saturation level in hyperglycemia. Overproduced ROS/RNS were recognized as important factors that divert glycolytic flux from ATP generation toward the formation of advanced glycation end products (AGEs), sorbitol, fructose diacylglycerol, and UDP-N-acetylglucosamine (UDP-GlcNAc) (Chung et al., 2003). As important nonmitochondrial sources of ROS/RNS, activated subpathways further enhance the generation of reactive oxygen free radical species (Singh et al., 2022).

### Chronic kidney hypoxia

Diabetes has previously been shown to induce “pseudohypoxia”, which refers to a state with increased lactate formation regardless of subsequent exposure to normoxic oxygen levels (Williamson et al., 1993). Later, studies confirmed the presence of intrarenal hypoxia in both cortical and medullary regions of diabetic kidneys, with significantly reduced oxygen tension (Palm et al., 2004; Rosenberger et al., 2008; Laustsen et al., 2014; Valdés et al., 2021). Importantly, a decreased level of renal oxygenation was also found in patients with diabetes (Yin et al., 2012). Hypoxia is the result of a mismatch between oxygen delivery and oxygen demand. Specifically, in models of early diabetic kidney involvement, higher levels of renal blood perfusion and glomerular filtration rates render “primarily ischemic” damage unlikely. Therefore, chronic hypoxia in early diabetic kidneys is mainly related to augmented oxygen consumption rather than impaired oxygen delivery or blood flow (Blantz, 2014). The enhanced tubular reabsorption and increased mitochondrial uncoupling can partially explain the increase in oxygen utilization (Körner et al., 1994; Palm et al., 2003; Friederich et al., 2008). In addition, oxygen diffusion distances increase as the extracellular matrix accumulates between blood vessels and adjacent cells over time (Fine and Norman, 2008).

Hypoxia is an established driver of the metabolic switch from mitochondrial oxidative phosphorylation to anaerobic fermentation, which was first observed by Pasteur in the late 19th century (Nelson and Cox, 2005). The metabolic reprogramming process seems to involve cell-autonomous adaptation that maintains ATP levels in response to oxygen deficiency under hypoxic conditions (Chen et al., 2017). Studies showed increased pyruvate-to-lactate production concomitant with unaltered oxidative phosphorylation and activation of the poly pathway in streptozotocin-induced diabetic kidneys when there is sufficient oxygen (Palm et al., 2004; Laustsen et al., 2013). Later, an experiment performed by Laustsen et al. further demonstrated an increased sensitivity of early diabetic kidneys to reduced oxygen availability and acquisition of a phenotype consistent with Warburg metabolism (Laustsen et al., 2014).

## Pathological damage induced by metabolic reprogramming of different cells in DKD

**FIGURE 2:**
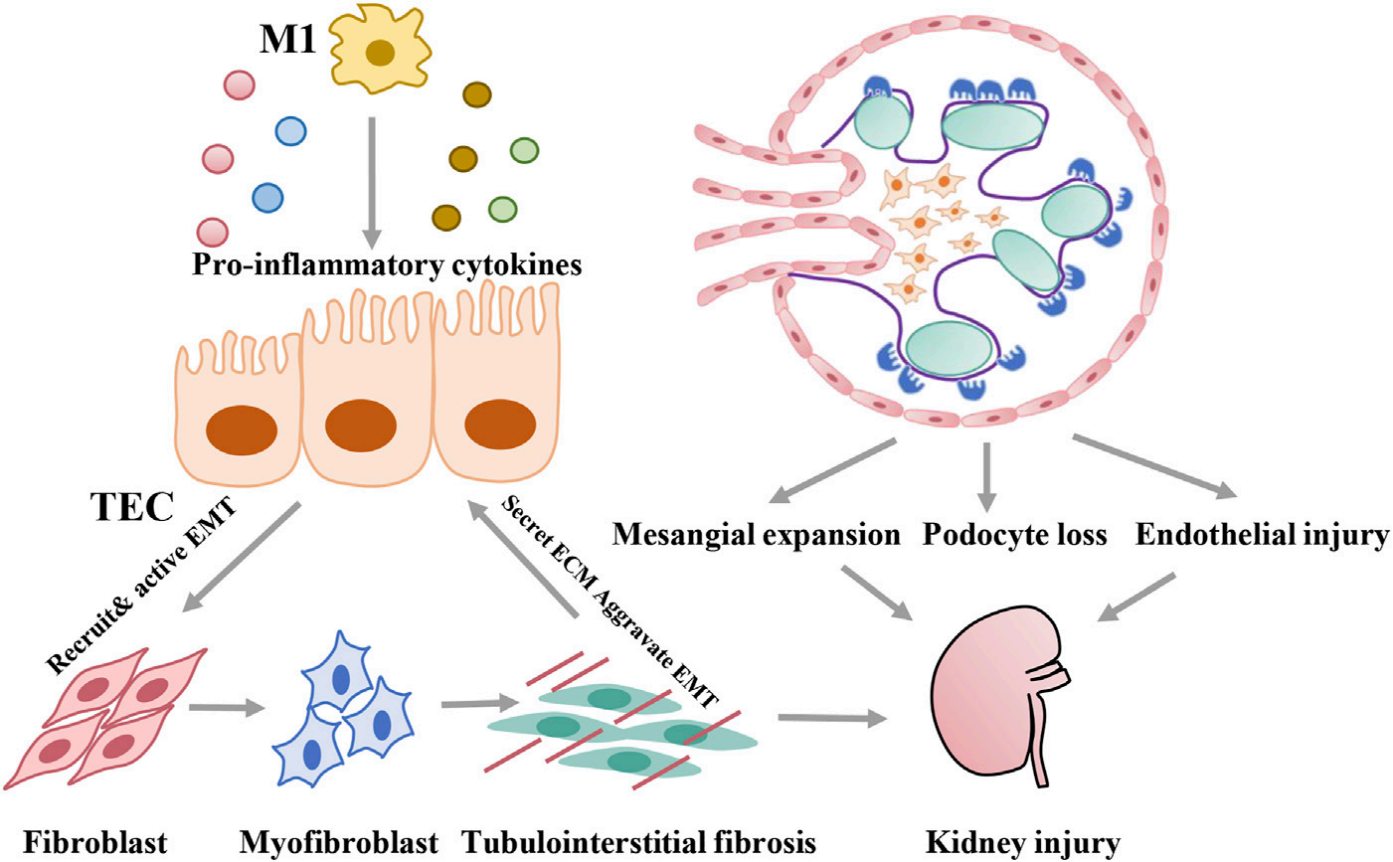
Pathological damage induced by metabolic reprogramming of different cells during development of DKD.

DKD is associated with structural changes that manifest as mesangial expansion, podocyte loss, tubular atrophy, and interstitial inflammation, which result in glomerulosclerosis and tubular interstitial fibrosis. Proximal tubular epithelial cells, with high-energy demands to enable constant reabsorption of nutrients, carry abundant mitochondria that rely mostly on fatty acid (FA) oxidation for energy at the baseline and undergo little glycolysis (Marks et al., 2003; Cargill and Sims-Lucas, 2020). In contrast, glomerular cells, including podocytes, mesangial cells, and glomerular endothelial cells, depend mainly on glucose for fuel (Abe et al., 2010; Bhargava and Schnellmann, 2017; Harzandi et al., 2021). In addition to resident renal cells, metabolic reprogramming can characterize immune cells, such as macrophages, which are closely related to kidney injury in diabetes. As discussed below, metabolic reprogramming in diabetes can induce multiple types of damage, including lipid accumulation, metabolite toxicity, ROS activation, and inflammation. With specific bioenergetic properties, cellular activation states vary between cell types, which contribute to specific pathological changes in the development of DKD (Figure 2).

### Tubular epithelial cells

Tubulointerstitial fibrosis is recognized as the common pathway of chronic kidney disease progression to ESRD. Healthy renal tubular epithelial cells require high levels of baseline energy; however, the capillaries around renal tubules are relatively sparse compared with those near glomerular cells, limiting the oxygen supply to renal tubules (Li S. et al., 2021). Therefore, renal tubule epithelial cells are more vulnerable to metabolic abnormities under diabetic conditions. Under normal conditions, extracellular FAs are transported into cells mainly via several FA transporters, among which cluster of differentiation 36 (CD36) (Pepino et al., 2014; Glatz and Luiken, 2018) and fatty acid-binding proteins (FABPs) are two important transporters. CD36, a transmembrane protein belonging to the class B scavenger receptor family, is the major receptor mediating the binding and uptake of FAs in proximal tubular epithelial cells (Yang et al., 2017). FABPs constitute a family of intracellular proteins that function in long-chain fatty acid (LCFA) uptake, metabolism, and intracellular transport in the cytoplasm. With 15 members discovered to date, FABP1 is expressed in epithelial tubular cells (Atshaves et al., 2010; Wang H. et al., 2021). After entering cells, LCFAs can be converted into LCFA-CoAs under the catalysis of acyl-CoA synthetase (ACS). Through FABP1 and carnitine shuttles, involving carnitine palmitoyltransferase-1 (CPT1) and CPT2 on the mitochondrial membrane, LCFAs and LCFA-CoAs are transported into the mitochondrial matrix for β-oxidation, which provides the TCA cycle with acetyl-CoA (Marks et al., 2003). Excessive acetyl-CoA can be transported out of mitochondria via carnitine acetyltransferase (CACT), which resynthesizes new FAs (Chen et al., 2019; Thongnak et al., 2020). Unconsumed FAs are converted to triglycerides and then into lipid droplets through two sequential reactions catalyzed by lipin-1 (LPIN1) and perlipin-2 (PLIN2) (Donkor et al., 2009). Recently, metabolic reprogramming in proximal tubular epithelial cells was demonstrated in both human and animal models (Czajka and Malik, 2016; Srivastava et al., 2018; Cai et al., 2020). On one hand, alterations in fuel-source preferences, from FAs to glucose, lead to impaired FA oxidation (FAO) in proximal tubular epithelial cells in the context of diabetes or sustained hyperglycemia. With the increased uptake of intracellular FAs (Su et al., 2017; Puchałowicz and Rać, 2020), excessive lipid droplets accumulate inside proximal tubular epithelial cells (Herman-Edelstein et al., 2014), which triggers further lipotoxicity by inducing inflammation, oxidative stress, endoplasmic reticulum stress, and so on and ultimately leads to cell apoptosis and renal fibrosis (Wang H. et al., 2021). On the other hand, many studies showed that the elevated expression of glycolytic enzymes and enhanced glycolysis in diabetes further induce epithelial–mesenchymal transition (EMT) and exacerbate renal fibrosis (Storch and Corsico, 2008); (Yin et al., 2018; Li et al., 2020a) In addition, several metabolites accumulate in the TCA cycle due to decreased mitochondrial mechanisms; one of these metabolites, fumarate, was shown to play a negative role in the mesenchymal activation (Sciacovelli et al., 2016)and cell death (Laustsen et al., 2020)of tubular epithelial cells in diabetic kidneys (You et al., 2016; Miura et al., 2019).

### Podocytes

Podocytes are highly specialized cells with complex structures known as interdigitating foot processes, slit diaphragms, and focal adhesion complexes, contributing to the formation of a glomerular filtration barrier. To sustain the complex cellular morphology as well as their normal function, podocytes rely on a constant energy supply involving both mitochondrial oxidative phosphorylation and glycolysis (Imasawa and Rossignol, 2013). The podocyte bioenergetic status seems to be dependent on their stage of differentiation. For example, aerobic glycolysis has been shown to be the main source of energy before differentiation, and OXPHOS is predominant during and after differentiation, with concomitant stimulation of mitochondrial biogenesis and functions (Imasawa et al., 2017; Yuan et al., 2020). In differentiated podocytes, metabolism switches to anaerobic glycolysis when the mitochondrial function is suppressed (Abe et al., 2010; Brinkkoetter et al., 2019). However, despite the significant decrease in mitochondrial oxidative phosphorylation as a result of PPARγ coactivator-1α (PGC-1α) and mitochondrial transcription factor A (TFAM) activity knockdown, no changes were found in urinary albumin excretion or glomerular morphology (Brinkkoetter et al., 2019). Therefore, the compensatory increase in glycolysis is thought to provide sufficient energy to meet podocyte needs under normal conditions; however, in the setting of cell stress such as hyperglycemia, the compensatory mechanism may not meet cellular needs. Metabolic reprogramming in HG-exposed human podocytes was shown to shift during a dedifferentiation process with decreased expression of functional proteins, such as podocin (Imasawa et al., 2017), leading to podocyte injury (Saleem et al., 2002). Further studies showed that inhibiting pyruvate from glycolysis to the TCA cycle in diabetic mice led to substantial podocyte damage, manifesting as a decrease in the number of cells and a reduction of synaptopodin (Qi W. et al., 2017; Li et al., 2020b). The increased flux to side branches of glycolysis is another mechanism of podocyte damage in metabolic reprogramming, instead of generating pyruvate, glucose enters side branches to produce toxic metabolites, such as sorbitol, methylglyoxal, and diacylglycerol, contributing to podocyte apoptosis (Qi et al., 2018). In addition, lipotoxicity in podocytes due to decreased metabolism has recently attracted attention (Audzeyenka et al., 2022). For example, fructose was shown to drive mitochondrial metabolic reprogramming in differentiated podocytes, resulting in lipid accumulation and cell injury (Fang et al., 2021).

### Mesangial cells

Glomerular mesangial cells (MCs) are specialized pericytes located around the glomerular capillaries within the renal corpuscle, and they synthesize the mesangial matrix and regulate glomerular hemodynamics via cell contraction and release various cytokines (Ebefors et al., 2021).Mesangial cell proliferation is stimulated in the early stage of DKD; subsequently, the growth of the cells is arrested, and they undergo hypertrophy and apoptosis (Khera et al., 2006; Tsai et al., 2020; Chen et al., 2022), contributing to glomerular sclerosis and a decline in the glomerular filtration rate. MCs exhibited higher basal respiration rates and reserved energy-production capacity, possibly making them more resistant to hyperglycemia. Their mitochondrial respiration was unaltered under hyperglycemic conditions for a short time. However, exposure to sustained hyperglycemia did not enhance glycolysis in MCs despite compromised mitochondrial respiration, in contrast to the effect on proximal tubular epithelial cells. Chronic hyperglycemia caused MCs to lose metabolic switching flexibility in response to an acutely high glucose load causing bioenergetic deficits in these cells (Czajka and Malik, 2016). An earlier study by Asano et al. (Asano et al., 2000) may have explained this phenomenon by showing that excessive glucose entered the sorbitol pathway, not the glycolytic pathway, in hyperglycemia, resulting in the accumulation of sorbitol and fructose in MCs. As a result, mesangial cells lost their contractile responsiveness and proliferative capacity (Derylo et al., 1998). Notably, a recent study by Xu et al. (Xu et al., 2021) revealed that glucose fluctuation, which refers to intermittent hyperglycemia, intensified aerobic glycolysis and suppressed OXPHOS in MCs, and suppressing the aerobic glycolytic switch improved cell viability, relieved inflammatory injury, and decreased the apoptosis rate.

### Endothelial cells

Glomerular endothelial cells (GECs), which reside within the glomerular capillary and are facilitated by fenestrae and a luminal glycocalyx layer, contribute to the formation of the glomerular filtration barrier (Haraldsson and Nyström, 2012). GEC dysfunction was recently intensively studied and was found to be a key perpetrator in the initiation and development of DKD (Fu J. et al., 2015; Shi and Vanhoutte, 2017; Maestroni and Zerbini, 2018). In contrast to other renal cells, endothelial cells primarily rely on glycolysis, not mitochondrial oxidative phosphorylation, for ATP production despite access to oxygen (Eelen et al., 2018). However, mitochondrial respiration still plays an important role in maintaining endothelial cell structural and functional integrity, such as by maintaining Ca^2+^ homeostasis and regulating oxidative stress (Yu et al., 2017; Yamamoto et al., 2018; Salnikova et al., 2021).

Diabetes is, in particular, a state of chronic hypoxia, and with elevated glucose uptake and disrupted glucose flow, it contributes to metabolic reprogramming in endothelial cells by further enhancing glycolysis and reducing mitochondrial respiratory capacity (Wu et al., 2017; Li J. et al., 2021; Dumas et al., 2021). Studies showed that hyperglycemia led to an upregulation of glycolytic metabolism and a downregulation of mitochondrial activity in GECs (Cheng et al., 2011; Qi H. et al., 2017; Song et al., 2022). Suppressed mitochondrial activity was associated with increased endothelin-1 receptor type A (EDNRA) expression and circulating endothelin-1 (ET-1) abundance, which led to the loss of fenestrae in GECs (Qi H. et al., 2017). Glycolytic activation promoted endothelial inflammation and macrophage infiltration (Song et al., 2022). In addition, a significant increase in dysfunction pathways can lead to increased oxidative stress. For example, enhanced glucose metabolism in the hexosamine pathway increased O-linked β-N-acetylglucosamine (O-GlcNAc) modification of endothelial nitric oxide synthase (eNOS) in experimental DKD and subsequent ROS production (Du et al., 2001). Excessive mitochondrial superoxide produced in dysfunctional mitochondria further increased side branch pathway metabolism (Clyne, 2021). Moreover, the metabolic response was accompanied by a series of molecular changes, such as increased expression of FASN (encodes fatty acid synthase) and arginase II (which catalyzes the hydrolysis of l-arginine and l-ornithine) and decreased biosynthesis of hyaluronan, which induces lipid accumulation (Wahl et al., 2016), triggers eNOS uncoupling, and reduces glycocalyx production in GECs (Wang G. et al., 2020). All these changes accelerated and exacerbated diabetic glomerular lesions and progression.

### Macrophages

Macrophages, originating from monocytes in peripheral blood, are classified into two distinct subtypes, M1 and M2 macrophages. Under homeostatic conditions, the M2 macrophage anti-inflammatory phenotype is predominant and depends mainly on OXPHOS for ATP. In contrast, stimulated resident macrophages acquire a proinflammatory M1 phenotype, which leads to inflammatory activity and preferential glycolysis even under conditions of sufficient oxygen (Curi et al., 2017). Metabolic reprogramming from OXPHOS toward aerobic glycolysis has been proven to be a primary indicator and central regulator during inflammatory activation by rapidly providing quiescent macrophages with sufficient energy (El Kasmi and Stenmark, 2015). Therefore, macrophages exhibit uniquely high metabolic plasticity, which enables them to respond quickly to external stimuli, including hyperglycemic signals.

A recent study showed that M1 polarization was increased in the kidneys of diabetic mice, and the upregulation of glycolytic enzyme expression, as well as lactic acid production and glucose uptake, was observed in high-glucose-stimulated macrophages. As a result, increased proinflammatory cytokine production caused pathological damage in DKD. Macrophage infiltration into glomeruli and the interstitium are related to renal impairment in DKD. Activated M1 macrophages secrete inflammatory cytokines, contributing to renal pathological damage, such as mesangial cell proliferation, podocyte apoptosis, and renal fibrosis (Chow et al., 2004; You et al., 2013; Lin et al., 2022).

## Cellular and molecular regulators of metabolic reprogramming in diabetic kidney disease

As discussed above, chronic hypoxia, increased glycolytic flux, and mitochondrial dysfunction are potential mechanisms of metabolic reprogramming. Hypoxia-inducible factor 1α (HIF-1α) is a well-known nucleoprotein activated under hypoxic conditions. Pyruvate kinase M2 (PKM2) is a key enzyme in glycolytic activity, and sirtuin 3 (SIRT3) directly interacts with various mitochondrial proteins, playing a crucial role in regulating mitochondrial functions. Therefore, we focus on the regulatory mechanisms mediated by these three molecules in the metabolic reprogramming of DKD (Table 1).

**TABLE 1:** Molecular regulators of metabolic reprogramming in DKD.

Key regulators	Author	Year	Models *in vivo*	Models *in vitro*	Effect on metabolic reprogramming	Expression in DKD	Injuries	References
HIF-1α	Ting Cai et al.	2020	Human with diabetes	proximal tubule	Promote	↑	mitigate related tubulointerstitial injury;	Cai et al. (2020)
			CD-1mice + STZ	epithelial cells (PTCs)			renal fibrosis	
HIF-1α	Hanxu Zeng et al.	2020	streptozotocin (STZ)-induced diabetic C57BL/6 mice	(HG)-stimulated bone marrow-derived macrophages (BMMs)	Promote	↑	renal inflammation	Zeng et al. (2020)
HIF-1α	Wei-Long Xu et al.	2021	-	The mouse glomerular mesangial cells (MCs)	Promote	↑	inflammation injury; apoptosis	Xu et al. (2021)
HIF-1α	Ryoichi Bessho et al.	2019	male db/db mice	human renal proximal tubular epithelial cells (HRPTECs)	Promote	↑	tubulointerstitial fibrosis	Bessho et al. (2019)
HIF-1α	Bijaya K. Nayak et al.	2016	OVE26 mice	Mesangial cells (MCs)	Promote	↑	glomerular injury;	Nayak et al. (2016)
							tubulointerstitial fibrosis	
HIF-1α	Keiichiro Matoba et al.	2013	male db/db mice	Murine mesangial cells (MES-13)	Promote	↑	glomerulosclerosis	Matoba et al. (2013)
PKM2	Weier Qi et al.	2017	Human with diabetes;	Mouse podocytes and human podocyte cell lines	Dimeric PKM2—Promote	Dimeric PKM2—↑	fibrosis in both glomeruli and tubules	Qi et al. (2017b)
			STZ-induced diabetic DBA2/J mice; diabetic eNos KO mice.			Tetrameric PKM2—↓		
PKM2	Le Li et al.	2020	db/db mice	HUVECs	Dimeric PKM2—Promote	Dimeric PKM2—↑	renal inflammation	Li et al. (2020c)
						Tetrameric PKM2—↓		
PKM2	Haijie Liu et al.	2021	CD-1 mice with STZ-induced diabetes	HK2 cells	Dimeric PKM2—Promote	Dimeric PKM2—↑	kidney fibrosis	Liu et al. (2021)
						Tetrameric PKM2—↓		
PKM2	Swayam Prakash Srivastava et al.	2018	CD-1 mice with STZ-induced diabetes;	-	Dimeric PKM2—Promote	Dimeric PKM2—↑	kidney fibrosis	Srivastava et al. (2018)
						Tetrameric PKM2—↓		
PKM2	Eva M Palsson-McDermott et al.	2015	-	BMDMs and PECs isolated from C57BL/6 mice	Dimeric PKM2—Promote	Dimeric PKM2—↑	inflammation	Palsson-McDermott et al. (2015)
						Tetrameric PKM2—↓		
PKM2	Jialin Fu	2022	STZ-induced diabetes; mice with PKM2 overexpression in podocytes (PPKM2Tg)	-	Dimeric PKM2—Promote	Dimeric PKM2—↑	fibrosis; inflammation	Fu et al. (2022)
						Tetrameric PKM2—↓		
SIRT3	Swayam Prakash Srivastava et al.	2021	CD-1 mice with STZ-induced diabetes	HMVECs; HK-2 cells	Suppress	↓	endothelial-to-mesenchymal transition; kidney fibrosis	Srivastava et al. (2021)
SIRT3	Jinpeng Li et al.	2020	CD-1 mice with STZ-induced diabetes	HK-2 proximal tubule cells; HMVECs.	Suppress	↓	epithelial-to-mesenchymal transition; endothelial-to-mesenchymal transition; kidney fibrosis	Li et al. (2020a)
SIRT3	Yunfei Wang et al.	2019	-	HUVECs	Suppress	↓	endothelial cell apoptosis in kidneys; renal inflammation injury	Wang et al. (2019)
SIRT3	Zhiwen Liu et al.	2019	db/db mice	mouse proximal tubular cell line (BUMPT)	Suppress	↓	renal oxidative damage and cell apoptosis	Liu et al. (2019)
SIRT3	Xiaocui Jiao et al.	2016	-	HK-2 cell	Suppress	↓	oxidative stress; renal tubular cell apoptosis	Jiao et al. (2016)
SIRT3	Ying Wang et al.	2021	-	HK-2 cell	Suppress	↓	inhibition of autophagy	Wang et al. (2021b)
SIRT3	Monica Locatelli et al.	2020	BTBR ob/ob mice with type 2 diabetes.	-	Suppress	↓	glomerular inflammation	Locatelli et al. (2020)
SIRT3	Li Zhuo et al.	2011	-	Rat mesangial cell line (MCs)	Suppress	↓	mesangial hypertrophy	Zhuo et al. (2011)

### HIF-1α

HIF-1α is the active subunit of HIF-1 and functions as a master regulator of cellular and systemic homeostatic responses to cytoplasmic hypoxia. Under normoxic conditions, HIF-1α is rapidly degraded through the ubiquitin–proteasome pathway, followed by hydroxylation by prolyl hydroxylases (PHDs). When the oxygen supply is limited, the increased stability of the active subunit leads to HIF-1α accumulation and translocation to the nucleus, where it binds to hypoxia response elements (HREs), resulting in elevated transcription of the target genes to facilitate metabolic adaptation to hypoxia (Wang et al., 1995).

HIF-1α is a key transcriptional regulator of metabolic modification. On one hand, HIF-1α reprograms central metabolism by enhancing glycolysis. HIF-1α functions as a direct transcriptional activator of the glucose transporters GLUT1 and GLUT3 and nearly all glycolytic enzymes, including phosphoglycerate kinase 1 (PGK-1), glucose-6-phosphate isomerase (GPI), phosphofructose kinase-1 (PFK-1), and lactate dehydrogenase (LDH), to promote both the uptake and catabolism of glucose (Hu et al., 2006; Zhong et al., 2010; Yan et al., 2017). On the other hand, HIF-1α negatively regulates mitochondrial respiration. Evidence suggests that HIF-1α suppresses the TCA cycle and ETC activity by preventing substrates, such as glucose and FAs, from being catabolized to acetyl-CoA, downregulating mitochondrial mass by promoting mitophagy and inhibiting mitochondrial biogenesis (Thomas and Ashcroft, 2019). In turn, mitochondrial dysfunction increases the levels of ROS, which can stabilize HIF-1α by inhibiting the activity of PHDs, and another oxygen-dependent dioxygenase enzyme, factor inhibiting HIF (FIH), promotes HIF degradation (Hagen, 2012). An increase in TCA cycle metabolites, such as succinate and fumarate, also leads to HIF-1α accumulation by inhibiting PHDs (Selak et al., 2005; You et al., 2016). Importantly, these processes progress independent of hypoxic. Additionally, HIF-1α signaling inhibits the diversion of pyruvate from glycolysis into the TCA cycle by increasing the expression of LDH and pyruvate dehydrogenase kinase (PDK), which phosphorylates and inactivates PDC (Kim et al., 2006).

A HIF-1α-mediated switching to glycolysis was observed in rodent models of DKD, proximal tubules, mesangial cells, and macrophages under HG conditions and proved to play a pivotal role in the fibrosis process of DKD by inducing inflammation, lipid accumulation, and the EMT (Matoba et al., 2013; Nayak et al., 2016; Bessho et al., 2019). Therefore, metabolic reprogramming regulated by HIF-1α is likely an important target for ameliorating DKD fibrosis.

### PKM2

PKM2 is a key isoform of pyruvate kinase (PK), acting as the rate-limiting glycolytic enzyme that catalyzes the final step from phosphoenolpyruvate (PEP) to pyruvate (Tsutsumi et al., 1988; Bluemlein et al., 2011). PKM2 is mainly expressed in the kidneys (Alquraishi et al., 2019), existing as an active tetramer, a less active dimer, or an inactive monomer (Wen et al., 2021). In most cases, PKM2 forms tetramers under physiological conditions, promoting the entry of pyruvate into the TCA cycle. However, researchers have identified a shift of PKM2 from tetramer to dimer or monomer formation in models of DKD (Sun et al., 2011; Qi W. et al., 2017; Li et al., 2020b), thereby shifting glucose metabolism toward aerobic glycolysis (Tamada et al., 2012). This shift to lower activity of PKM2 is always caused by post-translational modifications of PKM2, such as phosphorylation, acetylation, sulfenylation, and oxidation (Yang and Lu, 2015; Qi W. et al., 2017; Alquraishi et al., 2019).

Low-activity dimers or inactive monomers of PKM2 reduce the conversion of PEP to pyruvate, leading to accumulation of intermediary metabolites upstream. The intermediary metabolites are then available as precursors for the glycolytic side branches, leading to accumulation of toxic metabolites (Srivastava et al., 2018; Liu et al., 2021). Besides, the PKM2 dimer can be translocated into the nucleus via multiple mechanisms (Hitosugi et al., 2009; Yang et al., 2012; Wang et al., 2014; Yang and Lu, 2015), where it acts as a coactivator of HIF-1α (Luo et al., 2011) and signal transducer and activator of transcription 3 (STAT3) (Gao et al., 2012), a member of the STAT protein family, mainly in response to various cytokines and growth factors (You et al., 2015; Zheng et al., 2019) to promote metabolic reprogramming. Nuclear PKM2-mediated STAT3 has been reported to be sufficient to induce metabolic reprogramming of macrophages by increasing HIF-1 signaling (El Kasmi and Stenmark, 2015). The nuclear translocation of PKM2 was also shown to increase the expression of LDH and PDK1, thus ultimately leading to lactate accumulation (Luo et al., 2011; Yang and Lu, 2015). Moreover, researchers revealed that diabetic patients with advanced kidney functions had lower levels of active PKM2 in renal glomeruli and podocyte-specific PKM2-knockout (KO) mice with diabetes developed worse albuminuria and glomerular pathology. They further discovered that by activating PKM2, mitochondrial biogenesis, mitochondrial fusion, and mitochondrial membrane potential were re-established, suggesting that PKM2 also regulates metabolic reprogramming by affecting mitochondrial functions (Qi W. et al., 2017; Gordin et al., 2019). In addition, pathological PKM2 isoform switching has also been described in renal tubular epithelial cells and endothelial cells of diabetic kidneys, inducing inflammation by regulating intracellular metabolic reprogramming and, eventually, leading to glomerular lesions and renal fibrosis, which promotes DKD progression (Qi W. et al., 2017; Li L. et al., 2020; Liu et al., 2021).

### SIRT3

SIRT3, belonging to the sirtuin family, is a highly conserved nicotinamide adenine dinucleotide (NAD+)-dependent histone deacetylase (de Oliveira et al., 2010). It is expressed at high levels in the kidneys (Jin et al., 2009) as long or short isoforms (Murugasamy et al., 2022). The short isoform is predominant in the mitochondrial matrix, where it acts as a functionally active mitochondrial deacetylase (Onyango et al., 2002; Schwer et al., 2002). SIRT3 can directly interact with at least 84 mitochondrial proteins (Yang et al., 2016) and regulates several cellular processes, including mitochondrial DNA damage repair, gene expression, energy metabolism, redox balance, and autophagy (Ahn et al., 2008; Sundaresan et al., 2008; Cimen et al., 2010; Cheng et al., 2013; Li Y. et al., 2018).

However, with aging and under pathological conditions, SIRT3 expression is downregulated (Benigni et al., 2016). In a high-glucose environment, reduced SIRT3 abundance promotes glycolysis and inhibits OXPHOS through the regulation of oxidative stress and mitochondria-related proteases, resulting in metabolic reprogramming in kidney cells. Reduced SIRT3 levels directly inhibit the activity of manganese superoxide dismutase (MnSOD), which is the first line of defense against oxidative stress (Finley et al., 2011a). By inhibiting the deacetylation of its target protein forkhead box protein O3a (FOXO3a), SIRT3 depletion leads to the decreased activity of other antioxidants, such as catalase and isocitrate dehydrogenase 2 (IDH2), which is associated with glutathione reductase (Jacobs et al., 2008; Sundaresan et al., 2009; Yu et al., 2012). As a result, excessive ROS accumulates and stabilizes HIF-1α, which subsequently promotes the glycolytic process (Finley et al., 2011a). Studying SIRT3-knockout mice, researchers found hyperacetylation and reduced activity of enzymes involved in the TCA cycle and ETC activity, including NADH dehydrogenase ubiquinone 1 alpha subcomplex 9 (NDUFA9) in complex I, succinate dehydrogenase subunit A (SDHA) in complex II, and complex III (Ahn et al., 2008; Finley et al., 2011b; Wang S. et al., 2020). In addition, decreased SIRT3 levels could result in reduced mitochondrial biosynthesis, abnormal mitochondrial dynamics, impaired mitophagy, and an increase in abnormal mitochondria, ultimately contributing to mitochondrial dysfunction (Tseng et al., 2013; Feng et al., 2018). In addition, the lack of SIRT3 for promoting glycolysis has been associated with a higher PKM2 dimer formation rate (Srivastava et al., 2018) and activated STAT3 signaling (Srivastava et al., 2020b). Reduced SIRT3 levels lead to hyperacetylation and decreased PDC activity, thereby promoting glycolysis–glucose oxidative uncoupling and the accumulation of pyruvate/lactate (Bause and Haigis, 2013; Zhang et al., 2020).

## Emerging therapeutics for regulating metabolic reprogramming in diabetic kidney disease

Several strategies have been proven effective in controlling the metabolic switching between mitochondrial OXPHOS and glycolysis. Renin-angiotensin-aldosterone system (RAAS) inhibitors, including angiotensin-converting enzyme inhibitors (ACEIs) and angiotensin II receptor blockers (ARBs), are conventional therapies for DKD. A recent study by Srivastava et al. (Srivastava et al., 2020a) showed that imidapril, an ACE inhibitor, suppressed abnormal glucose metabolism through glycolysis and simultaneously restored mitochondrial FAO, thus ameliorating renal fibrosis in diabetic mice. The underlying mechanism might be related to the restoration of the expression of N-acetyl-seryl-aspartyl-lysyl-proline (AcSDKP), an endogenous peptide that is normally present in the plasma, and the exogenous addition of this peptide led to a similar effect. However, ARBs did not exert any effect on metabolic reprogramming.

Recently, large placebo-controlled studies confirmed the beneficial effects of SGLT2 inhibitors in delaying the progression of ESRD in diabetic patients (Neal et al., 2017; Perkovic et al., 2019). These inhibitors reduced tubular reabsorption of glucose, thus lowering blood glucose and enhancing renal oxygenation of the cortical region (Hesp et al., 2020). They also normalized TCA cycle activity, mitigated TCA-metabolite accumulation, and inhibited oxidative stress in the kidneys of diabetic mice (Tanaka et al., 2018). Recent studies suggested that dapagliflozin reduces diabetes-induced tubulointerstitial damage by suppressing metabolic switching from lipid oxidation to glycolysis (Cai et al., 2020), and another SGLT2 inhibitor, empagliflozin, was shown to protect kidney tubules from undergoing the EMT by normalizing suppressed SIRT3 levels and inhibiting aberrant glycolysis (Li et al., 2020a). In addition to the medicines already on the market, some preclinical therapies were also proven effective. For example, Interleukin-22 (IL-22), an endogenous cytokine secreted by immune cells, has been shown to correct metabolic reprogramming by maintaining mitochondrial integrity, reducing ROS, and inhibiting lipid accumulation in DKD (Chen et al., 2021; Shen et al., 2021). Glycolysis inhibitors and PKM2 activators have also been revealed to effectively disrupt metabolic reprogramming (Qi W. et al., 2017; Liu et al., 2021). Therefore, metabolic reprogramming is a pivotal target, and therapeutic strategies regulating metabolic reprogramming may be beneficial in retarding DKD progression.

## Conclusions and future perspectives

The regulation of the metabolic network is complicated. As we reviewed, a combination of several mechanisms accounts for metabolic reprogramming in DKD, and all these factors influence each other. Oxygen depletion directly leads to decreased mitochondrial metabolism and glycolytic pathway activation (Seagroves et al., 2001). In turn, increased mitochondrial uncoupling contributes to intrarenal hypoxia in diabetic kidneys by stimulating O_2_ consumption (Friederich et al., 2008). The increased side branches and mitochondria dysfunction interact with each other through ROS production (Brownlee, 2001). Thus, some key molecules of energy metabolism are discovered as core regulators of metabolic reprogramming and represent potential targets for the treatment, such as HIF-1α, PKM2, and SIRT3. An in-depth understanding of these key regulatory molecules will help to develop effective drugs to reverse energy metabolism abnormalities. Moreover, the affected cells are not isolated in the kidneys. There is also a cross-talk between cells, which means that a change in cellular metabolic reprogramming may cause damage to other cells. For example, the metabolic reprogramming of endothelial cells leads to podocyte defects and depletion (Qi H. et al., 2017). The elevated anaerobic glycolysis in renal tubular epithelial cells inhibited the proliferation and differentiation of co-incubated podocytes (Li M. et al., 2018). Therefore, future research should focus on metabolic reprogramming in more types of cells and the interaction between them.

In summary, the pathogenesis of DKD development is complex, and therapies that target a single mechanism or pathway show little effectiveness in treating the disease. Metabolic reprogramming includes multiple steps in energy molecule processing and can lead to a broad spectrum of abnormalities. Knowing the role played by metabolic reprogramming in DKD is of great importance for understanding the pathophysiology and opens the door to a variety of novel therapeutic applications.

## References

[B1] Abe Y. Sakairi T. Kajiyama H. Shrivastav S. Beeson C. Kopp J. B. (2010). Bioenergetic characterization of mouse podocytes. Am. J. Physiol. Cell Physiol. 299 (2), C464–C476. 10.1152/ajpcell.00563.2009 20445170 PMC2928644

[B2] Ahmad A. A. Draves S. O. Rosca M. (2021). Mitochondria in diabetic kidney disease. Cells 10 (11), 2945. 10.3390/cells10112945 34831168 PMC8616075

[B3] Ahn B. H. Kim H. S. Song S. Lee I. H. Liu J. Vassilopoulos A. (2008). A role for the mitochondrial deacetylase Sirt3 in regulating energy homeostasis. Proc. Natl. Acad. Sci. U. S. A. 105 (38), 14447–14452. 10.1073/pnas.0803790105 18794531 PMC2567183

[B4] Alicic R. Z. Rooney M. T. Tuttle K. R. (2017). Diabetic kidney disease: Challenges, progress, and possibilities. Clin. J. Am. Soc. Nephrol. 12 (12), 2032–2045. 10.2215/cjn.11491116 28522654 PMC5718284

[B5] Alquraishi M. Puckett D. L. Alani D. S. Humidat A. S. Frankel V. D. Donohoe D. R. (2019). Pyruvate kinase M2: A simple molecule with complex functions. Free Radic. Biol. Med. 143, 176–192. 10.1016/j.freeradbiomed.2019.08.007 31401304 PMC6848794

[B6] Asano T. Wakisaka M. Yoshinari M. Nakamura S. Doi Y. Fujishima M. (2000). Troglitazone enhances glycolysis and improves intracellular glucose metabolism in rat mesangial cells. Metabolism. 49 (3), 308–313. 10.1016/s0026-0495(00)90088-x 10726906

[B7] Atshaves B. P. Martin G. G. Hostetler H. A. McIntosh A. L. Kier A. B. Schroeder F. (2010). Liver fatty acid-binding protein and obesity. J. Nutr. Biochem. 21 (11), 1015–1032. 10.1016/j.jnutbio.2010.01.005 20537520 PMC2939181

[B8] Audzeyenka I. Bierżyńska A. Lay A. C. (2022). Podocyte bioenergetics in the development of diabetic nephropathy: The role of mitochondria. Endocrinology 163 (1), bqab234. 10.1210/endocr/bqab234 34791124 PMC8660556

[B9] Bause A. S. Haigis M. C. (2013). SIRT3 regulation of mitochondrial oxidative stress. Exp. Gerontol. 48 (7), 634–639. 10.1016/j.exger.2012.08.007 22964489

[B10] Benigni A. Perico L. Macconi D. (2016). Mitochondrial dynamics is linked to longevity and protects from end-organ injury: The emerging role of sirtuin 3. Antioxid. Redox Signal. 25 (4), 185–199. 10.1089/ars.2016.6682 26972664

[B11] Bessho R. Takiyama Y. Takiyama T. Kitsunai H. Takeda Y. Sakagami H. (2019). Hypoxia-inducible factor-1α is the therapeutic target of the SGLT2 inhibitor for diabetic nephropathy. Sci. Rep. 9 (1), 14754. 10.1038/s41598-019-51343-1 31611596 PMC6791873

[B12] Bhargava P. Schnellmann R. G. (2017). Mitochondrial energetics in the kidney. Nat. Rev. Nephrol. 13 (10), 629–646. 10.1038/nrneph.2017.107 28804120 PMC5965678

[B13] Blantz R. C. (2014). Phenotypic characteristics of diabetic kidney involvement. Kidney Int. 86 (1), 7–9. 10.1038/ki.2013.552 24978373 PMC4076684

[B14] Bluemlein K. Grüning N. M. Feichtinger R. G. Lehrach H. Kofler B. Ralser M. (2011). No evidence for a shift in pyruvate kinase PKM1 to PKM2 expression during tumorigenesis. Oncotarget 2 (5), 393–400. 10.18632/oncotarget.278 21789790 PMC3248187

[B15] Brady N. R. Hamacher-Brady A. Westerhoff H. V. Gottlieb R. A. (2006). A wave of reactive oxygen species (ROS)-induced ROS release in a sea of excitable mitochondria. Antioxid. Redox Signal. 8 (9-10), 1651–1665. 10.1089/ars.2006.8.1651 16987019

[B16] Brinkkoetter P. T. Bork T. Salou S. Liang W. Mizi A. Özel C. (2019). Anaerobic glycolysis maintains the glomerular filtration barrier independent of mitochondrial metabolism and dynamics. Cell Rep. 27 (5), 1551–1566. e1555. 10.1016/j.celrep.2019.04.012 31042480 PMC6506687

[B17] Brownlee M. (2001). Biochemistry and molecular cell biology of diabetic complications. Nature 414 (6865), 813–820. 10.1038/414813a 11742414

[B18] Buse M. G. (2006). Hexosamines, insulin resistance, and the complications of diabetes: Current status. Am. J. Physiol. Endocrinol. Metab. 290 (1), E1-E8–e8. 10.1152/ajpendo.00329.2005 16339923 PMC1343508

[B19] Cai T. Ke Q. Fang Y. Wen P. Chen H. Yuan Q. (2020). Sodium-glucose cotransporter 2 inhibition suppresses HIF-1α-mediated metabolic switch from lipid oxidation to glycolysis in kidney tubule cells of diabetic mice. Cell Death Dis. 11 (5), 390. 10.1038/s41419-020-2544-7 32444604 PMC7242894

[B20] Cargill K. Sims-Lucas S. (2020). Metabolic requirements of the nephron. Pediatr. Nephrol. 35 (1), 1–8. 10.1007/s00467-018-4157-2 30554363

[B21] Chen C. Shi Y. Ma J. Chen Z. Zhang M. Zhao Y. (2022). Trigonelline reverses high glucose-induced proliferation, fibrosis of mesangial cells via modulation of Wnt signaling pathway. Diabetol. Metab. Syndr. 14 (1), 28. 10.1186/s13098-022-00798-w 35139912 PMC8827266

[B22] Chen L. Duan Y. Wei H. Ning H. Bi C. Zhao Y. (2019). Acetyl-CoA carboxylase (ACC) as a therapeutic target for metabolic syndrome and recent developments in ACC1/2 inhibitors. Expert Opin. Investig. Drugs 28 (10), 917–930. 10.1080/13543784.2019.1657825 31430206

[B23] Chen W. Shen Y. Fan J. Zeng X. Zhang X. Luan J. (2021). IL-22-mediated renal metabolic reprogramming via PFKFB3 to treat kidney injury. Clin. Transl. Med. 11 (2), e324. 10.1002/ctm2.324 33634980 PMC7901723

[B24] Chen Y. Fry B. C. Layton A. T. (2017). Modeling glucose metabolism and lactate production in the kidney. Math. Biosci. 289, 116–129. 10.1016/j.mbs.2017.04.008 28495544 PMC5533195

[B25] Chen Y. Fry B. C. Layton A. T. (2016). Modeling glucose metabolism in the kidney. Bull. Math. Biol. 78 (6), 1318–1336. 10.1007/s11538-016-0188-7 27371260 PMC5431085

[B26] Cheng X. Siow R. C. Mann G. E. (2011). Impaired redox signaling and antioxidant gene expression in endothelial cells in diabetes: A role for mitochondria and the nuclear factor-E2-related factor 2-Kelch-like ECH-associated protein 1 defense pathway. Antioxid. Redox Signal. 14 (3), 469–487. 10.1089/ars.2010.3283 20524845

[B27] Cheng Y. Ren X. Gowda A. S. Shan Y. Zhang L. Yuan Y. S. (2013). Interaction of Sirt3 with OGG1 contributes to repair of mitochondrial DNA and protects from apoptotic cell death under oxidative stress. Cell Death Dis. 4 (7), e731. 10.1038/cddis.2013.254 23868064 PMC3730425

[B28] Chow F. Y. Nikolic-Paterson D. J. Atkins R. C. Tesch G. H. (2004). Macrophages in streptozotocin-induced diabetic nephropathy: Potential role in renal fibrosis. Nephrol. Dial. Transpl. 19 (12), 2987–2996. 10.1093/ndt/gfh441 15574996

[B29] Chung S. S. Ho E. C. Lam K. S. Chung S. K. (2003). Contribution of polyol pathway to diabetes-induced oxidative stress. J. Am. Soc. Nephrol. 14 (8), S233–S236. 10.1097/01.asn.0000077408.15865.06 12874437

[B30] Cimen H. Han M. J. Yang Y. Tong Q. Koc H. Koc E. C. (2010). Regulation of succinate dehydrogenase activity by SIRT3 in mammalian mitochondria. Biochemistry 49 (2), 304–311. 10.1021/bi901627u 20000467 PMC2826167

[B31] Clyne A. M. (2021). Endothelial response to glucose: Dysfunction, metabolism, and transport. Biochem. Soc. Trans. 49 (1), 313–325. 10.1042/bst20200611 33522573 PMC7920920

[B32] Curi R. de Siqueira Mendes R. de Campos Crispin L. A. Norata G. D. Sampaio S. C. Newsholme P. (2017). A past and present overview of macrophage metabolism and functional outcomes. Clin. Sci. 131 (12), 1329–1342. 10.1042/cs20170220 28592702

[B33] Czajka A. Ajaz S. Gnudi L. Parsade C. K. Jones P. Reid F. (2015). Altered mitochondrial function, mitochondrial DNA and reduced metabolic flexibility in patients with diabetic nephropathy. EBioMedicine 2 (6), 499–512. 10.1016/j.ebiom.2015.04.002 26288815 PMC4534759

[B34] Czajka A. Malik A. N. (2016). Hyperglycemia induced damage to mitochondrial respiration in renal mesangial and tubular cells: Implications for diabetic nephropathy. Redox Biol. 10, 100–107. 10.1016/j.redox.2016.09.007 27710853 PMC5053113

[B35] Dai W. Lu H. Chen Y. Yang D. Sun L. He L. (2021). The loss of mitochondrial quality control in diabetic kidney disease. Front. Cell Dev. Biol. 9, 706832. 10.3389/fcell.2021.706832 34422828 PMC8375501

[B36] de Oliveira R. M. Pais T. F. Outeiro T. F. (2010). Sirtuins: Common targets in aging and in neurodegeneration. Curr. Drug Targets 11 (10), 1270–1280. 10.2174/1389450111007011270 20840069

[B37] Derylo B. Babazono T. Glogowski E. Kapor-Drezgic J. Hohman T. Whiteside C. (1998). High glucose-induced mesangial cell altered contractility: Role of the polyol pathway. Diabetologia 41 (5), 507–515. 10.1007/s001250050939 9628266

[B38] Dlamini Z. Ntlabati P. Mbita Z. Shoba-Zikhali L. (2015). Pyruvate dehydrogenase kinase 4 (PDK4) could be involved in a regulatory role in apoptosis and a link between apoptosis and insulin resistance. Exp. Mol. Pathol. 98 (3), 574–584. 10.1016/j.yexmp.2015.03.022 25794976

[B39] Donkor J. Zhang P. Wong S. O'Loughlin L. Dewald J. Kok B. P. (2009). A conserved serine residue is required for the phosphatidate phosphatase activity but not the transcriptional coactivator functions of lipin-1 and lipin-2. J. Biol. Chem. 284 (43), 29968–29978. 10.1074/jbc.M109.023663 19717560 PMC2785625

[B40] Du X. L. Edelstein D. Dimmeler S. Ju Q. Sui C. Brownlee M. (2001). Hyperglycemia inhibits endothelial nitric oxide synthase activity by posttranslational modification at the Akt site. J. Clin. Invest. 108 (9), 1341–1348. 10.1172/jci11235 11696579 PMC209429

[B41] Dugan L. L. You Y. H. Ali S. S. Diamond-Stanic M. Miyamoto S. DeCleves A. E. (2013). AMPK dysregulation promotes diabetes-related reduction of superoxide and mitochondrial function. J. Clin. Invest. 123 (11), 4888–4899. 10.1172/jci66218 24135141 PMC3809777

[B42] Dumas S. J. Meta E. Borri M. Luo Y. Li X. Rabelink T. J. (2021). Phenotypic diversity and metabolic specialization of renal endothelial cells. Nat. Rev. Nephrol. 17 (7), 441–464. 10.1038/s41581-021-00411-9 33767431 PMC7993417

[B43] Ebefors K. Bergwall L. Nyström J. (2021). The glomerulus according to the mesangium. Front. Med. 8, 740527. 10.3389/fmed.2021.740527 PMC8825785 35155460

[B44] Eelen G. de Zeeuw P. Treps L. Harjes U. Wong B. W. Carmeliet P. (2018). Endothelial cell metabolism. Physiol. Rev. 98 (1), 3–58. 10.1152/physrev.00001.2017 29167330 PMC5866357

[B45] El Kasmi K. C. Stenmark K. R. (2015). Contribution of metabolic reprogramming to macrophage plasticity and function. Semin. Immunol. 27 (4), 267–275. 10.1016/j.smim.2015.09.001 26454572 PMC4677817

[B46] Elsas L. J. Longo N. (1992). Glucose transporters. Annu. Rev. Med. 43, 377–393. 10.1146/annurev.me.43.020192.002113 1580597

[B47] Fang L. Li T. S. Zhang J. Z. Liu Z. H. Yang J. Wang B. H. (2021). Fructose drives mitochondrial metabolic reprogramming in podocytes via Hmgcs2-stimulated fatty acid degradation. Signal Transduct. Target. Ther. 6 (1), 253. 10.1038/s41392-021-00570-y 34238920 PMC8266798

[B48] Feng J. Lu C. Dai Q. Sheng J. Xu M. (2018). SIRT3 facilitates amniotic fluid stem cells to repair diabetic nephropathy through protecting mitochondrial homeostasis by modulation of mitophagy. Cell. Physiol. biochem. 46 (4), 1508–1524. 10.1159/000489194 29689547

[B49] Fine L. G. Norman J. T. (2008). Chronic hypoxia as a mechanism of progression of chronic kidney diseases: From hypothesis to novel therapeutics. Kidney Int. 74 (7), 867–872. 10.1038/ki.2008.350 18633339

[B50] Finley L. W. Carracedo A. Lee J. Souza A. Egia A. Zhang J. (2011a). SIRT3 opposes reprogramming of cancer cell metabolism through HIF1α destabilization. Cancer Cell 19 (3), 416–428. 10.1016/j.ccr.2011.02.014 21397863 PMC3065720

[B51] Finley L. W. Haas W. Desquiret-Dumas V. Wallace D. C. Procaccio V. Gygi S. P. (2011b). Succinate dehydrogenase is a direct target of sirtuin 3 deacetylase activity. PLoS One 6 (8), e23295. 10.1371/journal.pone.0023295 21858060 PMC3157345

[B52] Friederich M. Fasching A. Hansell P. Nordquist L. Palm F. (2008). Diabetes-induced up-regulation of uncoupling protein-2 results in increased mitochondrial uncoupling in kidney proximal tubular cells. Biochim. Biophys. Acta 1777 (7-8), 935–940. 10.1016/j.bbabio.2008.03.030 18439413

[B53] Fu J. Lee K. Chuang P. Y. Liu Z. He J. C. (2015a). Glomerular endothelial cell injury and cross talk in diabetic kidney disease. Am. J. Physiol. Ren. Physiol. 308 (4), F287–F297. 10.1152/ajprenal.00533.2014 PMC4329492 25411387

[B54] Fu J. Shinjo T. Li Q. St-Louis R. Park K. Yu M. G. (2022). Regeneration of glomerular metabolism and function by podocyte pyruvate kinase M2 in diabetic nephropathy. JCI Insight 7 (5), e155260. 10.1172/jci.insight.155260 35133981 PMC8983139

[B55] Fu X. Chin R. M. Vergnes L. Hwang H. Deng G. Xing Y. (2015b). 2-Hydroxyglutarate inhibits ATP synthase and mTOR signaling. Cell Metab. 22 (3), 508–515. 10.1016/j.cmet.2015.06.009 26190651 PMC4663076

[B56] Gao X. Wang H. Yang J. J. Liu X. Liu Z. R. (2012). Pyruvate kinase M2 regulates gene transcription by acting as a protein kinase. Mol. Cell 45 (5), 598–609. 10.1016/j.molcel.2012.01.001 22306293 PMC3299833

[B57] Glatz J. F. C. Luiken J. (2018). Dynamic role of the transmembrane glycoprotein CD36 (SR-B2) in cellular fatty acid uptake and utilization. J. Lipid Res. 59 (7), 1084–1093. 10.1194/jlr.R082933 29627764 PMC6027920

[B58] Gordin D. Shah H. Shinjo T. St-Louis R. Qi W. Park K. (2019). Characterization of glycolytic enzymes and pyruvate kinase M2 in type 1 and 2 diabetic nephropathy. Diabetes Care 42 (7), 1263–1273. 10.2337/dc18-2585 31076418 PMC6609957

[B59] Group W. (2003). Sustained effect of intensive treatment of type 1 diabetes mellitus on development and progression of diabetic nephropathy: The epidemiology of diabetes interventions and complications (EDIC) study. Jama 290 (16), 2159–2167. 10.1001/jama.290.16.2159 14570951 PMC2622725

[B60] Hagen T. (2012). Oxygen versus reactive oxygen in the regulation of HIF-1α: The balance tips. Biochem. Res. Int. 2012, 436981. 10.1155/2012/436981 23091723 PMC3474226

[B61] Haraldsson B. Nyström J. (2012). The glomerular endothelium: New insights on function and structure. Curr. Opin. Nephrol. Hypertens. 21 (3), 258–263. 10.1097/MNH.0b013e3283522e7a 22388551

[B62] Harzandi A. Lee S. Bidkhori G. Saha S. Hendry B. M. Mardinoglu A. (2021). Acute kidney injury leading to CKD is associated with a persistence of metabolic dysfunction and hypertriglyceridemia. iScience 24 (2), 102046. 10.1016/j.isci.2021.102046 33554059 PMC7843454

[B63] Haynes R. Lewis D. Emberson J. Reith C. Agodoa L. Cass A. (2014). Effects of lowering LDL cholesterol on progression of kidney disease. J. Am. Soc. Nephrol. 25 (8), 1825–1833. 10.1681/asn.2013090965 24790178 PMC4116066

[B64] Heilig C. W. Brosius F. C. 3rd Henry D. N. (1997). Glucose transporters of the glomerulus and the implications for diabetic nephropathy. Kidney Int. Suppl. 60, S91–S99. 9285909

[B65] Herman-Edelstein M. Scherzer P. Tobar A. Levi M. Gafter U. (2014). Altered renal lipid metabolism and renal lipid accumulation in human diabetic nephropathy. J. Lipid Res. 55 (3), 561–572. 10.1194/jlr.P040501 24371263 PMC3934740

[B66] Hesp A. C. Schaub J. A. Prasad P. V. Vallon V. Laverman G. D. Bjornstad P. (2020). The role of renal hypoxia in the pathogenesis of diabetic kidney disease: A promising target for newer renoprotective agents including SGLT2 inhibitors? Kidney Int. 98 (3), 579–589. 10.1016/j.kint.2020.02.041 32739206 PMC8397597

[B67] Higgins G. C. Coughlan M. T. (2014). Mitochondrial dysfunction and mitophagy: The beginning and end to diabetic nephropathy? Br. J. Pharmacol. 171 (8), 1917–1942. 10.1111/bph.12503 24720258 PMC3976613

[B68] Hitosugi T. Kang S. Vander Heiden M. G. Chung T. W. Elf S. Lythgoe K. (2009). Tyrosine phosphorylation inhibits PKM2 to promote the Warburg effect and tumor growth. Sci. Signal. 2 (97), ra73. 10.1126/scisignal.2000431 19920251 PMC2812789

[B69] Hu C. J. Iyer S. Sataur A. Covello K. L. Chodosh L. A. Simon M. C. (2006). Differential regulation of the transcriptional activities of hypoxia-inducible factor 1 alpha (HIF-1alpha) and HIF-2alpha in stem cells. Mol. Cell. Biol. 26 (9), 3514–3526. 10.1128/mcb.26.9.3514-3526.2006 16611993 PMC1447431

[B70] Imasawa T. Obre E. Bellance N. Lavie J. Imasawa T. Rigothier C. (2017). High glucose repatterns human podocyte energy metabolism during differentiation and diabetic nephropathy. Faseb J. 31 (1), 294–307. 10.1096/fj.201600293R 27825100 PMC5161522

[B71] Imasawa T. Rossignol R. (2013). Podocyte energy metabolism and glomerular diseases. Int. J. Biochem. Cell Biol. 45 (9), 2109–2118. 10.1016/j.biocel.2013.06.013 23806869

[B72] Jacobs K. M. Pennington J. D. Bisht K. S. Aykin-Burns N. Kim H. S. Mishra M. (2008). SIRT3 interacts with the daf-16 homolog FOXO3a in the mitochondria, as well as increases FOXO3a dependent gene expression. Int. J. Biol. Sci. 4 (5), 291–299. 10.7150/ijbs.4.291 18781224 PMC2532794

[B73] Jeoung N. H. Wu P. Joshi M. A. Jaskiewicz J. Bock C. B. Depaoli-Roach A. A. (2006). Role of pyruvate dehydrogenase kinase isoenzyme 4 (PDHK4) in glucose homoeostasis during starvation. Biochem. J. 397 (3), 417–425. 10.1042/bj20060125 16606348 PMC1533314

[B74] Jiang Y. K. Xin K. Y. Ge H. W. Kong F. J. Zhao G. (2019). Upregulation of renal GLUT2 and SGLT2 is involved in high-fat diet-induced gestational diabetes in mice. Diabetes Metab. Syndr. Obes. 12, 2095–2105. 10.2147/dmso.S221396 31686881 PMC6800457

[B75] Jiao X. Li Y. Zhang T. Liu M. Chi Y. (2016). Role of Sirtuin3 in high glucose-induced apoptosis in renal tubular epithelial cells. Biochem. Biophys. Res. Commun. 480 (3), 387–393. 10.1016/j.bbrc.2016.10.060 27773814

[B76] Jin L. Galonek H. Israelian K. Choy W. Morrison M. Xia Y. (2009). Biochemical characterization, localization, and tissue distribution of the longer form of mouse SIRT3. Protein Sci. 18 (3), 514–525. 10.1002/pro.50 19241369 PMC2760358

[B77] Karlstaedt A. Zhang X. Vitrac H. Harmancey R. Vasquez H. Wang J. H. (2016). Oncometabolite d-2-hydroxyglutarate impairs α-ketoglutarate dehydrogenase and contractile function in rodent heart. Proc. Natl. Acad. Sci. U. S. A. 113 (37), 10436–10441. 10.1073/pnas.1601650113 27582470 PMC5027422

[B78] Khera T. Martin J. Riley S. Steadman R. Phillips A. O. (2006). Glucose enhances mesangial cell apoptosis. Lab. Invest. 86 (6), 566–577. 10.1038/labinvest.3700418 16585941

[B79] Kim J. W. Tchernyshyov I. Semenza G. L. Dang C. V. (2006). HIF-1-mediated expression of pyruvate dehydrogenase kinase: A metabolic switch required for cellular adaptation to hypoxia. Cell Metab. 3 (3), 177–185. 10.1016/j.cmet.2006.02.002 16517405

[B80] Körner A. Eklöf A. C. Celsi G. Aperia A. Korner A. (1994). Increased renal metabolism in diabetes. Mechanism and functional implications. Diabetes 43 (5), 629–633. 10.2337/diab.43.5.629 8168637

[B81] Laustsen C. Lycke S. Palm F. Østergaard J. A. Bibby B. M. Nørregaard R. (2014). High altitude may alter oxygen availability and renal metabolism in diabetics as measured by hyperpolarized [1-(13)C]pyruvate magnetic resonance imaging. Kidney Int. 86 (1), 67–74. 10.1038/ki.2013.504 24352155

[B82] Laustsen C. Nielsen P. M. Qi H. Løbner M. H. Palmfeldt J. Bertelsen L. B. (2020). Hyperpolarized [1, 4-(13)C]fumarate imaging detects microvascular complications and hypoxia mediated cell death in diabetic nephropathy. Sci. Rep. 10 (1), 9650. 10.1038/s41598-020-66265-6 32541797 PMC7295762

[B83] Laustsen C. Østergaard J. A. Lauritzen M. H. Nørregaard R. Bowen S. Søgaard L. V. (2013). Assessment of early diabetic renal changes with hyperpolarized [1-(13) C]pyruvate. Diabetes. Metab. Res. Rev. 29 (2), 125–129. 10.1002/dmrr.2370 23166087

[B84] Lewko B. Bryl E. Witkowski J. M. Latawiec E. Angielski S. Stepinski J. (2005). Mechanical stress and glucose concentration modulate glucose transport in cultured rat podocytes. Nephrol. Dial. Transpl. 20 (2), 306–311. 10.1093/ndt/gfh612 15673689

[B85] Li J. Fang Y. Wu D. (2021a). Mechanical forces and metabolic changes cooperate to drive cellular memory and endothelial phenotypes. Curr. Top. Membr. 87, 199–253. 10.1016/bs.ctm.2021.07.003 34696886 PMC8639155

[B86] Li J. Liu H. Takagi S. Nitta K. Kitada M. Srivastava S. P. (2020a). Renal protective effects of empagliflozin via inhibition of EMT and aberrant glycolysis in proximal tubules. JCI Insight 5 (6), 129034. 10.1172/jci.insight.129034 32134397 PMC7213787

[B87] Li J. Sun Y. B. Y. Chen W. Fan J. Li S. Qu X. (2020b). Smad4 promotes diabetic nephropathy by modulating glycolysis and OXPHOS. EMBO Rep. 21 (2), e48781. 10.15252/embr.201948781 31916354 PMC7001498

[B88] Li L. Tang L. Yang X. Chen R. Zhang Z. Leng Y. (2020c). Gene regulatory effect of pyruvate kinase M2 is involved in renal inflammation in type 2 diabetic nephropathy. Exp. Clin. Endocrinol. Diabetes 128 (9), 599–606. 10.1055/a-1069-7290 31958846

[B89] Li M. Jia F. Zhou H. Di J. Yang M. (2018a). Elevated aerobic glycolysis in renal tubular epithelial cells influences the proliferation and differentiation of podocytes and promotes renal interstitial fibrosis. Eur. Rev. Med. Pharmacol. Sci. 22 (16), 5082–5090. 10.26355/eurrev_201808_15701 30178826

[B90] Li S. Wang F. Sun D. (2021b). The renal microcirculation in chronic kidney disease: Novel diagnostic methods and therapeutic perspectives. Cell Biosci. 11 (1), 90. 10.1186/s13578-021-00606-4 34001267 PMC8130426

[B91] Li Y. Ma Y. Song L. Yu L. Zhang L. Zhang Y. (2018b). SIRT3 deficiency exacerbates p53/Parkin-mediated mitophagy inhibition and promotes mitochondrial dysfunction: Implication for aged hearts. Int. J. Mol. Med. 41 (6), 3517–3526. 10.3892/ijmm.2018.3555 29532856

[B92] Lin X. Lei X. Q. Yang J. K. Jia J. Zhong X. Tan R. Z. (2022). Astragalus mongholicus Bunge and Panax notoginseng formula (A&P) improves renal mesangial cell damage in diabetic nephropathy by inhibiting the inflammatory response of infiltrated macrophages. BMC Complement. Med. Ther. 22 (1), 17. 10.1186/s12906-021-03477-x 35057768 PMC8781170

[B93] Liu H. Takagaki Y. Kumagai A. Kanasaki K. Koya D. (2021). The PKM2 activator TEPP-46 suppresses kidney fibrosis via inhibition of the EMT program and aberrant glycolysis associated with suppression of HIF-1α accumulation. J. Diabetes Investig. 12 (5), 697–709. 10.1111/jdi.13478 PMC8089020 33314682

[B94] Liu Z. Liu H. Xiao L. Liu G. Sun L. He L. (2019). STC-1 ameliorates renal injury in diabetic nephropathy by inhibiting the expression of BNIP3 through the AMPK/SIRT3 pathway. Lab. Invest. 99 (5), 684–697. 10.1038/s41374-018-0176-7 30683904

[B95] Locatelli M. Zoja C. Zanchi C. Corna D. Villa S. Bolognini S. (2020). Manipulating Sirtuin 3 pathway ameliorates renal damage in experimental diabetes. Sci. Rep. 10 (1), 8418. 10.1038/s41598-020-65423-0 32439965 PMC7242337

[B96] Luo W. Hu H. Chang R. Zhong J. Knabel M. O'Meally R. (2011). Pyruvate kinase M2 is a PHD3-stimulated coactivator for hypoxia-inducible factor 1. Cell 145 (5), 732–744. 10.1016/j.cell.2011.03.054 21620138 PMC3130564

[B97] Maestroni S. Zerbini G. (2018). Glomerular endothelial cells versus podocytes as the cellular target in diabetic nephropathy. Acta Diabetol. 55 (11), 1105–1111. 10.1007/s00592-018-1211-2 30155580

[B98] Marks J. Carvou N. J. Debnam E. S. Srai S. K. Unwin R. J. (2003). Diabetes increases facilitative glucose uptake and GLUT2 expression at the rat proximal tubule brush border membrane. J. Physiol. 553 (1), 137–145. 10.1113/jphysiol.2003.046268 12963802 PMC2343472

[B99] Mather A. Pollock C. (2011). Glucose handling by the kidney. Kidney Int. Suppl (120), S1–S6. 10.1038/ki.2010.509 21358696

[B100] Matoba K. Kawanami D. Okada R. Tsukamoto M. Kinoshita J. Ito T. (2013). Rho-kinase inhibition prevents the progression of diabetic nephropathy by downregulating hypoxia-inducible factor 1α. Kidney Int. 84 (3), 545–554. 10.1038/ki.2013.130 23615507

[B101] Miura Y. Hayakawa A. Kikuchi S. Tsumoto H. Umezawa K. Chiba Y. (2019). Fumarate accumulation involved in renal diabetic fibrosis in Goto-Kakizaki rats. Arch. Biochem. Biophys. 678, 108167. 10.1016/j.abb.2019.108167 31704098

[B102] Moutzouris D. A. Kitsiou P. V. Talamagas A. A. Drossopoulou G. I. Kassimatis T. I. Katsilambros N. K. (2007). Chronic exposure of human glomerular epithelial cells to high glucose concentration results in modulation of high-affinity glucose transporters expression. Ren. Fail. 29 (3), 353–358. 10.1080/08860220601184126 17497451

[B103] Murugasamy K. Munjal A. Sundaresan N. R. (2022). Emerging roles of SIRT3 in cardiac metabolism. Front. Cardiovasc. Med. 9, 850340. 10.3389/fcvm.2022.850340 35369299 PMC8971545

[B104] Nayak B. K. Shanmugasundaram K. Friedrichs W. E. Cavaglierii R. C. Patel M. Barnes J. (2016). HIF-1 mediates renal fibrosis in OVE26 type 1 diabetic mice. Diabetes 65 (5), 1387–1397. 10.2337/db15-0519 26908870 PMC4839204

[B105] Neal B. Perkovic V. Matthews D. R. (2017). Canagliflozin and cardiovascular and renal events in type 2 diabetes. N. Engl. J. Med. 377 (21), 2098. 10.1056/NEJMc1712572 29166232

[B106] Nelson D. L. Cox M. M. (2005). Lehninger principles of biochemistry. 4th ed.

[B107] Onyango P. Celic I. McCaffery J. M. Boeke J. D. Feinberg A. P. (2002). SIRT3, a human SIR2 homologue, is an NAD-dependent deacetylase localized to mitochondria. Proc. Natl. Acad. Sci. U. S. A. 99 (21), 13653–13658. 10.1073/pnas.222538099 12374852 PMC129731

[B108] Pagliarini R. Podrini C. (2021). Metabolic reprogramming and reconstruction: Integration of experimental and computational studies to set the path forward in ADPKD. Front. Med. 8, 740087. 10.3389/fmed.2021.740087 PMC8652061 34901057

[B109] Palm F. Cederberg J. Hansell P. Liss P. Carlsson P. O. (2003). Reactive oxygen species cause diabetes-induced decrease in renal oxygen tension. Diabetologia 46 (8), 1153–1160. 10.1007/s00125-003-1155-z 12879251

[B110] Palm F. Hansell P. Ronquist G. Waldenström A. Liss P. Carlsson P. O. (2004). Polyol-pathway-dependent disturbances in renal medullary metabolism in experimental insulin-deficient diabetes mellitus in rats. Diabetologia 47 (7), 1223–1231. 10.1007/s00125-004-1434-3 15232683

[B111] Palsson-McDermott E. M. Curtis A. M. Goel G. Lauterbach M. A. Sheedy F. J. Gleeson L. E. (2015). Pyruvate kinase M2 regulates Hif-1α activity and IL-1β induction and is a critical determinant of the warburg effect in LPS-activated macrophages. Cell Metab. 21 (1), 65–80. 10.1016/j.cmet.2014.12.005 25565206 PMC5198835

[B112] Pepino M. Y. Kuda O. Samovski D. Abumrad N. A. (2014). Structure-function of CD36 and importance of fatty acid signal transduction in fat metabolism. Annu. Rev. Nutr. 34, 281–303. 10.1146/annurev-nutr-071812-161220 24850384 PMC4329921

[B113] Perkovic V. Jardine M. J. Neal B. Bompoint S. Heerspink H. J. L. Charytan D. M. (2019). Canagliflozin and renal outcomes in type 2 diabetes and nephropathy. N. Engl. J. Med. 380 (24), 2295–2306. 10.1056/NEJMoa1811744 30990260

[B114] Puchałowicz K. Rać M. E. (2020). The multifunctionality of CD36 in diabetes mellitus and its complications-update in pathogenesis, treatment and monitoring. Cells 9 (8), E1877. 10.3390/cells9081877 32796572 PMC7465275

[B115] Qi H. Casalena G. Shi S. Yu L. Ebefors K. Sun Y. (2017a). Glomerular endothelial mitochondrial dysfunction is essential and characteristic of diabetic kidney disease susceptibility. Diabetes 66 (3), 763–778. 10.2337/db16-0695 27899487 PMC5319717

[B116] Qi W. Keenan H. A. Li Q. Ishikado A. Kannt A. Sadowski T. (2017b). Pyruvate kinase M2 activation may protect against the progression of diabetic glomerular pathology and mitochondrial dysfunction. Nat. Med. 23 (6), 753–762. 10.1038/nm.4328 28436957 PMC5575773

[B117] Qi W. Li Q. Gordin D. King G. L. (2018). Preservation of renal function in chronic diabetes by enhancing glomerular glucose metabolism. J. Mol. Med. 96 (5), 373–381. 10.1007/s00109-018-1630-0 29574544 PMC7132623

[B118] Rardin M. J. Wiley S. E. Naviaux R. K. Murphy A. N. Dixon J. E. (2009). Monitoring phosphorylation of the pyruvate dehydrogenase complex. Anal. Biochem. 389 (2), 157–164. 10.1016/j.ab.2009.03.040 19341700 PMC2713743

[B119] Rosenberger C. Khamaisi M. Abassi Z. Shilo V. Weksler-Zangen S. Goldfarb M. (2008). Adaptation to hypoxia in the diabetic rat kidney. Kidney Int. 73 (1), 34–42. 10.1038/sj.ki.5002567 17914354

[B120] Saleem M. A. Ni L. Witherden I. Tryggvason K. Ruotsalainen V. Mundel P. (2002). Co-Localization of nephrin, podocin, and the actin cytoskeleton: Evidence for a role in podocyte foot process formation. Am. J. Pathol. 161 (4), 1459–1466. 10.1016/s0002-9440(10)64421-5 12368218 PMC1867300

[B121] Salnikova D. Orekhova V. Grechko A. Starodubova A. Bezsonov E. Popkova T. (2021). Mitochondrial dysfunction in vascular wall cells and its role in atherosclerosis. Int. J. Mol. Sci. 22 (16), 8990. 10.3390/ijms22168990 34445694 PMC8396504

[B122] Sas K. M. Kayampilly P. Byun J. Nair V. Hinder L. M. Hur J. (2016). Tissue-specific metabolic reprogramming drives nutrient flux in diabetic complications. JCI Insight 1 (15), e86976. 10.1172/jci.insight.86976 27699244 PMC5033761

[B123] Schwer B. North B. J. Frye R. A. Ott M. Verdin E. (2002). The human silent information regulator (Sir)2 homologue hSIRT3 is a mitochondrial nicotinamide adenine dinucleotide-dependent deacetylase. J. Cell Biol. 158 (4), 647–657. 10.1083/jcb.200205057 12186850 PMC2174009

[B124] Sciacovelli M. Gonçalves E. Johnson T. I. Zecchini V. R. da Costa A. S. Gaude E. (2016). Fumarate is an epigenetic modifier that elicits epithelial-to-mesenchymal transition. Nature 537 (7621), 544–547. 10.1038/nature19353 27580029 PMC5136292

[B125] Seagroves T. N. Ryan H. E. Lu H. Wouters B. G. Knapp M. Thibault P. (2001). Transcription factor HIF-1 is a necessary mediator of the pasteur effect in mammalian cells. Mol. Cell. Biol. 21 (10), 3436–3444. 10.1128/mcb.21.10.3436-3444.2001 11313469 PMC100265

[B126] Selak M. A. Armour S. M. MacKenzie E. D. Boulahbel H. Watson D. G. Mansfield K. D. (2005). Succinate links TCA cycle dysfunction to oncogenesis by inhibiting HIF-alpha prolyl hydroxylase. Cancer Cell 7 (1), 77–85. 10.1016/j.ccr.2004.11.022 15652751

[B127] Shen Y. Chen W. Han L. Bian Q. Fan J. Cao Z. (2021). VEGF-B antibody and interleukin-22 fusion protein ameliorates diabetic nephropathy through inhibiting lipid accumulation and inflammatory responses. Acta Pharm. Sin. B 11 (1), 127–142. 10.1016/j.apsb.2020.07.002 33532185 PMC7838033

[B128] Shi Y. Vanhoutte P. M. (2017). Macro- and microvascular endothelial dysfunction in diabetes. J. Diabetes 9 (5), 434–449. 10.1111/1753-0407.12521 28044409

[B129] Shiraishi T. Verdone J. E. Huang J. Kahlert U. D. Hernandez J. R. Torga G. (2015). Glycolysis is the primary bioenergetic pathway for cell motility and cytoskeletal remodeling in human prostate and breast cancer cells. Oncotarget 6 (1), 130–143. 10.18632/oncotarget.2766 25426557 PMC4381583

[B130] Singh A. Kukreti R. Saso L. Kukreti S. (2022). Mechanistic insight into oxidative stress-triggered signaling pathways and type 2 diabetes. Molecules 27 (3), 950. 10.3390/molecules27030950 35164215 PMC8840622

[B131] Song C. Wang S. Fu Z. Chi K. Geng X. Liu C. (2022). IGFBP5 promotes diabetic kidney disease progression by enhancing PFKFB3-mediated endothelial glycolysis. Cell Death Dis. 13 (4), 340. 10.1038/s41419-022-04803-y 35418167 PMC9007962

[B132] Srivastava S. P. Goodwin J. E. Kanasaki K. Koya D. (2020a). Metabolic reprogramming by N-acetyl-seryl-aspartyl-lysyl-proline protects against diabetic kidney disease. Br. J. Pharmacol. 177 (16), 3691–3711. 10.1111/bph.15087 32352559 PMC7393199

[B133] Srivastava S. P. Kanasaki K. Goodwin J. E. Koya D. (2020b). Inhibition of angiotensin-converting enzyme ameliorates renal fibrosis by mitigating DPP-4 level and restoring antifibrotic MicroRNAs. Genes 11, E211. 10.3390/genes11020211 32085655 PMC7074526

[B134] Srivastava S. P. Li J. Kitada M. Fujita H. Yamada Y. Goodwin J. E. (2018). SIRT3 deficiency leads to induction of abnormal glycolysis in diabetic kidney with fibrosis. Cell Death Dis. 9 (10), 997. 10.1038/s41419-018-1057-0 30250024 PMC6155322

[B135] Srivastava S. P. Li J. Takagaki Y. Kitada M. Goodwin J. E. Kanasaki K. (2021). Endothelial SIRT3 regulates myofibroblast metabolic shifts in diabetic kidneys. iScience 24 (5), 102390. 10.1016/j.isci.2021.102390 33981977 PMC8086030

[B136] Storch J. Corsico B. (2008). The emerging functions and mechanisms of mammalian fatty acid-binding proteins. Annu. Rev. Nutr. 28, 73–95. 10.1146/annurev.nutr.27.061406.093710 18435590

[B137] Su W. Cao R. He Y. C. Guan Y. F. Ruan X. Z. (2017). Crosstalk of hyperglycemia and dyslipidemia in diabetic kidney disease. Kidney Dis. 3 (4), 171–180. 10.1159/000479874 PMC5757547 29344511

[B138] Sun J. K. Keenan H. A. Cavallerano J. D. Asztalos B. F. Schaefer E. J. Sell D. R. (2011). Protection from retinopathy and other complications in patients with type 1 diabetes of extreme duration: The joslin 50-year medalist study. Diabetes Care 34 (4), 968–974. 10.2337/dc10-1675 21447665 PMC3064059

[B139] Sundaresan N. R. Gupta M. Kim G. Rajamohan S. B. Isbatan A. Gupta M. P. (2009). Sirt3 blocks the cardiac hypertrophic response by augmenting Foxo3a-dependent antioxidant defense mechanisms in mice. J. Clin. Invest. 119 (9), 2758–2771. 10.1172/jci39162 19652361 PMC2735933

[B140] Sundaresan N. R. Samant S. A. Pillai V. B. Rajamohan S. B. Gupta M. P. (2008). SIRT3 is a stress-responsive deacetylase in cardiomyocytes that protects cells from stress-mediated cell death by deacetylation of Ku70. Mol. Cell. Biol. 28 (20), 6384–6401. 10.1128/mcb.00426-08 18710944 PMC2577434

[B141] Tamada M. Suematsu M. Saya H. (2012). Pyruvate kinase M2: Multiple faces for conferring benefits on cancer cells. Clin. Cancer Res. 18 (20), 5554–5561. 10.1158/1078-0432.Ccr-12-0859 23071357

[B142] Tanaka S. Sugiura Y. Saito H. Sugahara M. Higashijima Y. Yamaguchi J. (2018). Sodium-glucose cotransporter 2 inhibition normalizes glucose metabolism and suppresses oxidative stress in the kidneys of diabetic mice. Kidney Int. 94 (5), 912–925. 10.1016/j.kint.2018.04.025 30021702

[B143] Thomas L. W. Ashcroft M. (2019). Exploring the molecular interface between hypoxia-inducible factor signalling and mitochondria. Cell. Mol. Life Sci. 76 (9), 1759–1777. 10.1007/s00018-019-03039-y 30767037 PMC6453877

[B144] Thongnak L. Pongchaidecha A. Lungkaphin A. (2020). Renal lipid metabolism and lipotoxicity in diabetes. Am. J. Med. Sci. 359 (2), 84–99. 10.1016/j.amjms.2019.11.004 32039770

[B145] Tsai Y. C. Kuo M. C. Hung W. W. Wu L. Y. Wu P. H. Chang W. A. (2020). High glucose induces mesangial cell apoptosis through miR-15b-5p and promotes diabetic nephropathy by extracellular vesicle delivery. Mol. Ther. 28 (3), 963–974. 10.1016/j.ymthe.2020.01.014 31991106 PMC7054723

[B146] Tseng A. H. Shieh S. S. Wang D. L. (2013). SIRT3 deacetylates FOXO3 to protect mitochondria against oxidative damage. Free Radic. Biol. Med. 63, 222–234. 10.1016/j.freeradbiomed.2013.05.002 23665396

[B147] Tsutsumi H. Tani K. Fujii H. Miwa S. (1988). Expression of L- and M-type pyruvate kinase in human tissues. Genomics 2 (1), 86–89. 10.1016/0888-7543(88)90112-7 2838416

[B148] Tuttle K. R. Bakris G. L. Bilous R. W. Chiang J. L. de Boer I. H. Goldstein-Fuchs J. (2014). Diabetic kidney disease: A report from an ADA consensus conference. Diabetes Care 37 (10), 2864–2883. 10.2337/dc14-1296 25249672 PMC4170131

[B149] Valdés A. Lucio-Cazaña F. J. Castro-Puyana M. García-Pastor C. Fiehn O. Marina M. L. (2021). Comprehensive metabolomic study of the response of HK-2 cells to hyperglycemic hypoxic diabetic-like milieu. Sci. Rep. 11 (1), 5058. 10.1038/s41598-021-84590-2 33658594 PMC7930035

[B150] Venditti P. Di Meo S. (2020). The role of reactive oxygen species in the life cycle of the mitochondrion. Int. J. Mol. Sci. 21 (6), E2173. 10.3390/ijms21062173 32245255 PMC7139706

[B151] Wahl P. Ducasa G. M. Fornoni A. (2016). Systemic and renal lipids in kidney disease development and progression. Am. J. Physiol. Ren. Physiol. 310 (6), F433–F445. 10.1152/ajprenal.00375.2015 PMC4971889 26697982

[B152] Wang G. Kostidis S. Tiemeier G. L. Sol W. de Vries M. R. Giera M. (2020a). Shear stress regulation of endothelial glycocalyx structure is determined by glucobiosynthesis. Arterioscler. Thromb. Vasc. Biol. 40 (2), 350–364. 10.1161/atvbaha.119.313399 31826652

[B153] Wang G. L. Jiang B. H. Rue E. A. Semenza G. L. (1995). Hypoxia-inducible factor 1 is a basic-helix-loop-helix-PAS heterodimer regulated by cellular O2 tension. Proc. Natl. Acad. Sci. U. S. A. 92 (12), 5510–5514. 10.1073/pnas.92.12.5510 7539918 PMC41725

[B154] Wang H. J. Hsieh Y. J. Cheng W. C. Lin C. P. Lin Y. S. Yang S. F. (2014). JMJD5 regulates PKM2 nuclear translocation and reprograms HIF-1α-mediated glucose metabolism. Proc. Natl. Acad. Sci. U. S. A. 111 (1), 279–284. 10.1073/pnas.1311249111 24344305 PMC3890888

[B155] Wang H. Zhang S. Guo J. (2021a). Lipotoxic proximal tubular injury: A primary event in diabetic kidney disease. Front. Med. 8, 751529. 10.3389/fmed.2021.751529 PMC8573085 34760900

[B156] Wang S. Zhang J. Deng X. Zhao Y. Xu K. (2020b). Advances in characterization of SIRT3 deacetylation targets in mitochondrial function. Biochimie 179, 1–13. 10.1016/j.biochi.2020.08.021 32898647

[B157] Wang Y. Chang J. Wang Z. Q. Li Y. (2021b). Sirt3 promotes the autophagy of HK-2 human proximal tubular epithelial cells via the inhibition of Notch-1/Hes-1 signaling. Mol. Med. Rep. 24 (3), 634. 10.3892/mmr.2021.12273 34278469 PMC8281085

[B158] Wang Y. Zhang X. Wang P. Shen Y. Yuan K. Li M. (2019). Sirt3 overexpression alleviates hyperglycemia-induced vascular inflammation through regulating redox balance, cell survival, and AMPK-mediated mitochondrial homeostasis. J. Recept. Signal Transduct. Res. 39 (4), 341–349. 10.1080/10799893.2019.1684521 31680596

[B159] Wang Z. Ying Z. Bosy-Westphal A. Zhang J. Schautz B. Later W. (2010). Specific metabolic rates of major organs and tissues across adulthood: Evaluation by mechanistic model of resting energy expenditure. Am. J. Clin. Nutr. 92 (6), 1369–1377. 10.3945/ajcn.2010.29885 20962155 PMC2980962

[B160] Warburg O. Wind F. Negelein E. (1927). The metabolism of tumors in the body. J. Gen. Physiol. 8 (6), 519–530. 10.1085/jgp.8.6.519 19872213 PMC2140820

[B161] Weigert C. Brodbeck K. Brosius F. C. 3rd Huber M. Lehmann R. Friess U. (2003). Evidence for a novel TGF-beta1-independent mechanism of fibronectin production in mesangial cells overexpressing glucose transporters. Diabetes 52 (2), 527–535. 10.2337/diabetes.52.2.527 12540631

[B162] Wen L. Li Y. Li S. Hu X. Wei Q. Dong Z. (2021). Glucose metabolism in acute kidney injury and kidney repair. Front. Med. 8, 744122. 10.3389/fmed.2021.744122 PMC8666949 34912819

[B163] Williamson J. R. Chang K. Frangos M. Hasan K. S. Ido Y. Kawamura T. (1993). Hyperglycemic pseudohypoxia and diabetic complications. Diabetes 42 (6), 801–813. 10.2337/diab.42.6.801 8495803

[B164] Wu D. Huang R. T. Hamanaka R. B. Krause M. Oh M. J. Kuo C. H. (2017). HIF-1α is required for disturbed flow-induced metabolic reprogramming in human and porcine vascular endothelium. Elife 6, e25217. 10.7554/eLife.25217 28556776 PMC5495571

[B165] Xu W. L. Liu S. Li N. Ye L. F. Zha M. Li C. Y. (2021). Quercetin antagonizes glucose fluctuation induced renal injury by inhibiting aerobic glycolysis via HIF-1α/miR-210/ISCU/FeS pathway. Front. Med. 8, 656086. 10.3389/fmed.2021.656086 PMC7969708 33748166

[B166] Yamamoto K. Imamura H. Ando J. (2018). Shear stress augments mitochondrial ATP generation that triggers ATP release and Ca(2+) signaling in vascular endothelial cells. Am. J. Physiol. Heart Circ. Physiol. 315 (5), H1477-H1485–h1485. 10.1152/ajpheart.00204.2018 30141983 PMC6297820

[B167] Yan J. Huang X. Zhu D. Lou Y. (2017). Enhanced aerobic glycolysis by S-nitrosoglutathione via HIF-1α associated GLUT1/aldolase A Axis in human endothelial cells. J. Cell. Biochem. 118 (8), 2443–2453. 10.1002/jcb.25911 28121054

[B168] Yang W. Lu Z. (2015). Pyruvate kinase M2 at a glance. J. Cell Sci. 128 (9), 1655–1660. 10.1242/jcs.166629 25770102 PMC4446733

[B169] Yang W. Nagasawa K. Münch C. Xu Y. Satterstrom K. Jeong S. (2016). Mitochondrial sirtuin network reveals dynamic SIRT3-dependent deacetylation in response to membrane depolarization. Cell 167 (4), 985–1000. 10.1016/j.cell.2016.10.016 27881304 PMC5134900

[B170] Yang W. Zheng Y. Xia Y. Ji H. Chen X. Guo F. (2012). ERK1/2-dependent phosphorylation and nuclear translocation of PKM2 promotes the Warburg effect. Nat. Cell Biol. 14 (12), 1295–1304. 10.1038/ncb2629 23178880 PMC3511602

[B171] Yang X. Okamura D. M. Lu X. Chen Y. Moorhead J. Varghese Z. (2017). CD36 in chronic kidney disease: Novel insights and therapeutic opportunities. Nat. Rev. Nephrol. 13 (12), 769–781. 10.1038/nrneph.2017.126 28919632

[B172] Yin W. J. Liu F. Li X. M. Yang L. Zhao S. Huang Z. X. (2012). Noninvasive evaluation of renal oxygenation in diabetic nephropathy by BOLD-MRI. Eur. J. Radiol. 81 (7), 1426–1431. 10.1016/j.ejrad.2011.03.045 21470811

[B173] Yin X. N. Wang J. Cui L. F. Fan W. X. (2018). Enhanced glycolysis in the process of renal fibrosis aggravated the development of chronic kidney disease. Eur. Rev. Med. Pharmacol. Sci. 22 (13), 4243–4251. 10.26355/eurrev_201807_15419 30024614

[B174] You H. Gao T. Cooper T. K. Brian Reeves W. Awad A. S. (2013). Macrophages directly mediate diabetic renal injury. Am. J. Physiol. Ren. Physiol. 305 (12), F1719–F1727. 10.1152/ajprenal.00141.2013 PMC3882451 24173355

[B175] You L. Wang Z. Li H. Shou J. Jing Z. Xie J. (2015). The role of STAT3 in autophagy. Autophagy 11 (5), 729–739. 10.1080/15548627.2015.1017192 25951043 PMC4509450

[B176] You Y. H. Quach T. Saito R. Pham J. Sharma K. (2016). Metabolomics reveals a key role for fumarate in mediating the effects of NADPH oxidase 4 in diabetic kidney disease. J. Am. Soc. Nephrol. 27 (2), 466–481. 10.1681/asn.2015030302 26203118 PMC4731129

[B177] Yu E. P. K. Reinhold J. Yu H. Starks L. Uryga A. K. Foote K. (2017). Mitochondrial respiration is reduced in atherosclerosis, promoting necrotic core formation and reducing relative fibrous cap thickness. Arterioscler. Thromb. Vasc. Biol. 37 (12), 2322–2332. 10.1161/atvbaha.117.310042 28970293 PMC5701734

[B178] Yu W. Dittenhafer-Reed K. E. Denu J. M. (2012). SIRT3 protein deacetylates isocitrate dehydrogenase 2 (IDH2) and regulates mitochondrial redox status. J. Biol. Chem. 287 (17), 14078–14086. 10.1074/jbc.M112.355206 22416140 PMC3340192

[B179] Yuan Q. Miao J. Yang Q. Fang L. Fang Y. Ding H. (2020). Role of pyruvate kinase M2-mediated metabolic reprogramming during podocyte differentiation. Cell Death Dis. 11 (5), 355. 10.1038/s41419-020-2481-5 32393782 PMC7214446

[B180] Zeng H. Qi X. Xu X. Wu Y. (2020). TAB1 regulates glycolysis and activation of macrophages in diabetic nephropathy. Inflamm. Res. 69 (12), 1215–1234. 10.1007/s00011-020-01411-4 33044562 PMC7658079

[B181] Zhang J. Xiang H. Liu J. Chen Y. He R. R. Liu B. (2020). Mitochondrial Sirtuin 3: New emerging biological function and therapeutic target. Theranostics 10 (18), 8315–8342. 10.7150/thno.45922 32724473 PMC7381741

[B182] Zhang L. Krzentowski G. Albert A. Lefèbvre P. J. (2003). Factors predictive of nephropathy in DCCT Type 1 diabetic patients with good or poor metabolic control. Diabet. Med. 20 (7), 580–585. 10.1046/j.1464-5491.2003.00986.x 12823241

[B183] Zhang P. N. Zhou M. Q. Guo J. Zheng H. J. Tang J. Zhang C. (2021). Mitochondrial dysfunction and diabetic nephropathy: Nontraditional therapeutic opportunities. J. Diabetes Res. 2021, 1010268. 10.1155/2021/1010268 34926696 PMC8677373

[B184] Zheng C. Huang L. Luo W. Yu W. Hu X. Guan X. (2019). Inhibition of STAT3 in tubular epithelial cells prevents kidney fibrosis and nephropathy in STZ-induced diabetic mice. Cell Death Dis. 10 (11), 848. 10.1038/s41419-019-2085-0 31699972 PMC6838321

[B185] Zhong L. D'Urso A. Toiber D. Sebastian C. Henry R. E. Vadysirisack D. D. (2010). The histone deacetylase Sirt6 regulates glucose homeostasis via Hif1alpha. Cell 140 (2), 280–293. 10.1016/j.cell.2009.12.041 20141841 PMC2821045

[B186] Zhu X. Jiang L. Long M. Wei X. Hou Y. Du Y. (2021). Metabolic reprogramming and renal fibrosis. Front. Med. 8, 746920. 10.3389/fmed.2021.746920 PMC8630632 34859009

[B187] Zhuo L. Fu B. Bai X. Zhang B. Wu L. Cui J. (2011). NAD blocks high glucose induced mesangial hypertrophy via activation of the sirtuins-AMPK-mTOR pathway. Cell. Physiol. biochem. 27 (6), 681–690. 10.1159/000330077 21691086

[B188] Zorov D. B. Juhaszova M. Sollott S. J. (2014). Mitochondrial reactive oxygen species (ROS) and ROS-induced ROS release. Physiol. Rev. 94 (3), 909–950. 10.1152/physrev.00026.2013 24987008 PMC4101632

[B189] Zorov D. B. Juhaszova M. Sollott S. J. (2006). Mitochondrial ROS-induced ROS release: An update and review. Biochim. Biophys. Acta 1757 (5-6), 509–517. 10.1016/j.bbabio.2006.04.029 16829228

